# Convergent application of traditional Chinese medicine and gut microbiota in ameliorate of cirrhosis: a data mining and Mendelian randomization study

**DOI:** 10.3389/fcimb.2023.1273031

**Published:** 2023-11-06

**Authors:** Cheng Zhou, Jingjing Wei, Peng Yu, Jinqiu Yang, Tong Liu, Ran Jia, Siying Wang, Pengfei Sun, Lin Yang, Haijuan Xiao

**Affiliations:** ^1^ The First College of Clinical Medicine, Henan University of Chinese Medicine, Zhengzhou, China; ^2^ Department of Orthopaedics, Jiangsu Province Hospital of Chinese Medicine, Nanjing, China; ^3^ Department of Hepatobiliary Surgery, Xianyang Central Hospital Affiliated to Shaanxi University of Chinese Medicine, Xianyang, China; ^4^ Department of Oncology, Affiliated Hospital of Shaanxi University of Chinese Medicine, Xianyang, China

**Keywords:** hepatitis B cirrhosis, traditional Chinese medicine, data mining, network analysis, gut microbiota, mendelian randomization

## Abstract

**Objective:**

Traditional Chinese medicine (TCM) has been used for the treatment of chronic liver diseases for a long time, with proven safety and efficacy in clinical settings. Previous studies suggest that the therapeutic mechanism of TCM for hepatitis B cirrhosis may involve the gut microbiota. Nevertheless, the causal relationship between the gut microbiota, which is closely linked to TCM, and cirrhosis remains unknown. This study aims to utilize two-sample Mendelian randomization (MR) to investigate the potential causal relationship between gut microbes and cirrhosis, as well as to elucidate the synergistic mechanisms between botanical drugs and microbiota in treating cirrhosis.

**Methods:**

Eight databases were systematically searched through May 2022 to identify clinical studies on TCM for hepatitis B cirrhosis. We analyzed the frequency, properties, flavors, and meridians of Chinese medicinals based on TCM theories and utilized the Apriori algorithm to identify the core botanical drugs for cirrhosis treatment. Cross-database comparison elucidated gut microbes sharing therapeutic targets with these core botanical drugs. MR analysis assessed consistency between gut microbiota causally implicated in cirrhosis and microbiota sharing therapeutic targets with key botanicals.

**Results:**

Our findings revealed differences between the Chinese medicinals used for compensated and decompensated cirrhosis, with distinct frequency, dosage, properties, flavors, and meridian based on TCM theory. *Angelicae Sinensis Radix, Salviae Miltiorrhizae Radix Et Rhizoma, Poria, Paeoniae Radix Alba, Astragali Radix, Atrctylodis Macrocephalae Rhizoma* were the main botanicals. Botanical drugs and gut microbiota target MAPK1, VEGFA, STAT3, AKT1, RELA, JUN, and ESR1 in the treatment of hepatitis B cirrhosis, and their combined use has shown promise for cirrhosis treatment. MR analysis demonstrated a positive correlation between increased ClostridialesvadinBB60 and Ruminococcustorques abundance and heightened cirrhosis risk. In contrast, Eubacteriumruminantium, Lachnospiraceae, Eubacteriumnodatum, RuminococcaceaeNK4A214, Veillonella, and RuminococcaceaeUCG002 associated with reduced cirrhosis risk. Notably, Lachnospiraceae shares key therapeutic targets with core botanicals, which can treat cirrhosis at a causal level.

**Conclusion:**

We identified 6 core botanical drugs for managing compensated and decompensated hepatitis B cirrhosis, despite slight prescription differences. The core botanical drugs affected cirrhosis through multiple targets and pathways. The shared biological effects between botanicals and protective gut microbiota offer a potential explanation for the therapeutic benefits of these key herbal components in treating cirrhosis. Elucidating these mechanisms provides crucial insights to inform new drug development and optimize clinical therapy for hepatitis B cirrhosis.

## Introduction

Cirrhosis is the end-stage of chronic liver diseases, including viral hepatitis, non-alcoholic steatohepatitis, autoimmune hepatitis, etc (Ginès et al., 2021). Globally, 30 percent of cirrhosis is caused by HBV infection (Tu and Douglas, 2020). Despite the inclusion of hepatitis B vaccine in China’s routine infant immunization program in 1992, the prevalence of hepatitis B in the general population has remained at about 6.89% in recent years (Wang et al., 2019). In a large-scale clinical research study, the probability of progression to hepatocellular carcinoma over a 10-year period in patients with inactive hepatitis B cirrhosis was about 32.48%, highlighting the need for individualized cirrhosis management beyond antivirals (Liu et al., 2021). TCM is the characteristic culture of ancient Chinese people for confronting disease, as well as a medical theory system progressively formed and developed through extensive medical practice under the guidance of simple materialism and spontaneous dialectic thought (Xutian et al., 2012). It is worth noting that TCM, which is regarded as an alternative therapy with clear efficacy, has long been used to treat chronic liver diseases (Daniyal et al., 2019). Nowadays, modern extraction and analysis of active compounds has further verified its clinical efficacy and safety (Wang et al., 2018; Wang et al., 2020).

Compensated hepatitis B cirrhosis represents the asymptomatic stage, in which patients have no obvious clinical symptoms, with weakness, abdominal distension, hepatosplenomegaly, liver palms and spider nevus as common manifestations (Ginès et al., 2021). Without effective treatment, complications such as ascites, hepatic encephalopathy, hepatorenal syndrome or variceal bleeding will rapidly arise, progressing to decompensated cirrhosis (Engelmann et al., 2021). Current standard management relies heavily on symptomatic medications, which do not reduce the high rates of hospitalization and mortality associated with hepatitis B cirrhosis. (Li et al., 2019; Li et al., 2021).

The clinical application of dialectical principles is fundamental to TCM therapy. According to studies, TCM is an effective way to halt cirrhosis progression and improve prognosis (Dong et al., 2015; Nan et al., 2022). TCM treatment of hepatitis B cirrhosis has gradually become a new trend. Rather than single herbs, TCM formulas comprise principal, ministerial, adjuvant, and messenger components to optimize treatment effects. However, TCM for hepatitis B cirrhosis requires the adjustment of prescriptions at different stages, causing the diversification of clinical usage, and a unified understanding of the medication rules has not yet been formed.

The liver and intestine share direct anatomical connections via the portal venous system and biliary tract, as well as indirect links through systemic circulation (Wang et al., 2022). Bacteria, bacterial products, and intestinal metabolites enter circulation and play a significant role in various liver diseases and complications associated with portal hypertension (Xu et al., 2022). In patients with cirrhosis, abnormalities in the immune, vascular, and inflammatory systems are linked to decreased intestinal microbiota function and compromised intestinal mucosal integrity (Trebicka et al., 2021). The pharmacological characteristics of Chinese medicinals and their compounds include multi-component, multi-target, and multi-pathway actions. The complex components of Chinese medicinals and their compounds can directly target specific areas while also adjusting bodily functions by affecting metabolic substances produced by gut microbiota. Intriguingly, some metabolites produced by probiotics have similar compositions or therapeutic targets as those found in botanical compounds. Consequently, when probiotic levels decline, supplementing with botanicals can achieve a similar effect to restoring probiotic levels.

Mendelian randomization (MR) is a method that uses genetic variation in single nucleotide polymorphisms (SNPs) as an instrumental variable (IV) to assess causal relationships between exposures and outcomes, overcoming biases in traditional observational studies (Ziegler et al., 2008; Yavorska and Burgess, 2017). Observational studies are limited by issues like confounding and reverse causation that yield biased results and hinder causal inference. However, MR can overcome these limitations because the probability of inheriting either allele is random and independent of confounders and reverse causation.

Systematic pharmacology is an effective method to combine traditional medicine with modern medicine. By analyzing the cross-targets and pathways of drugs and diseases, the biological mechanism of TCM in treating diseases can be completely described (Zhang et al., 2019). To conduct a comprehensive prescription analysis, we utilized data mining to collect data from 330 clinical studies of TCM for treating compensated/decompensated hepatitis B cirrhosis. Network analysis identified core botanical targets for cirrhosis, which were compared to gut microbial targets in the GutMGene Database (http://bio-annotation.cn/gutmgene/). Microbes sharing targets with core botanicals were identified. These botanicals may exert similar biological effects to the associated microbes for cirrhosis treatment. In turn, MR analysis was performed to confirm whether these microbes are causally associated with cirrhosis. The results suggest that microbes with similar biological actions to the botanicals are causally associated with the risk of cirrhosis, and that the 6 botanicals may play an important role by maintaining the biological actions of cirrhosis-associated gut microbiota.

## Materials and methods

### Datasets collection and analysis

**Figure 1:**
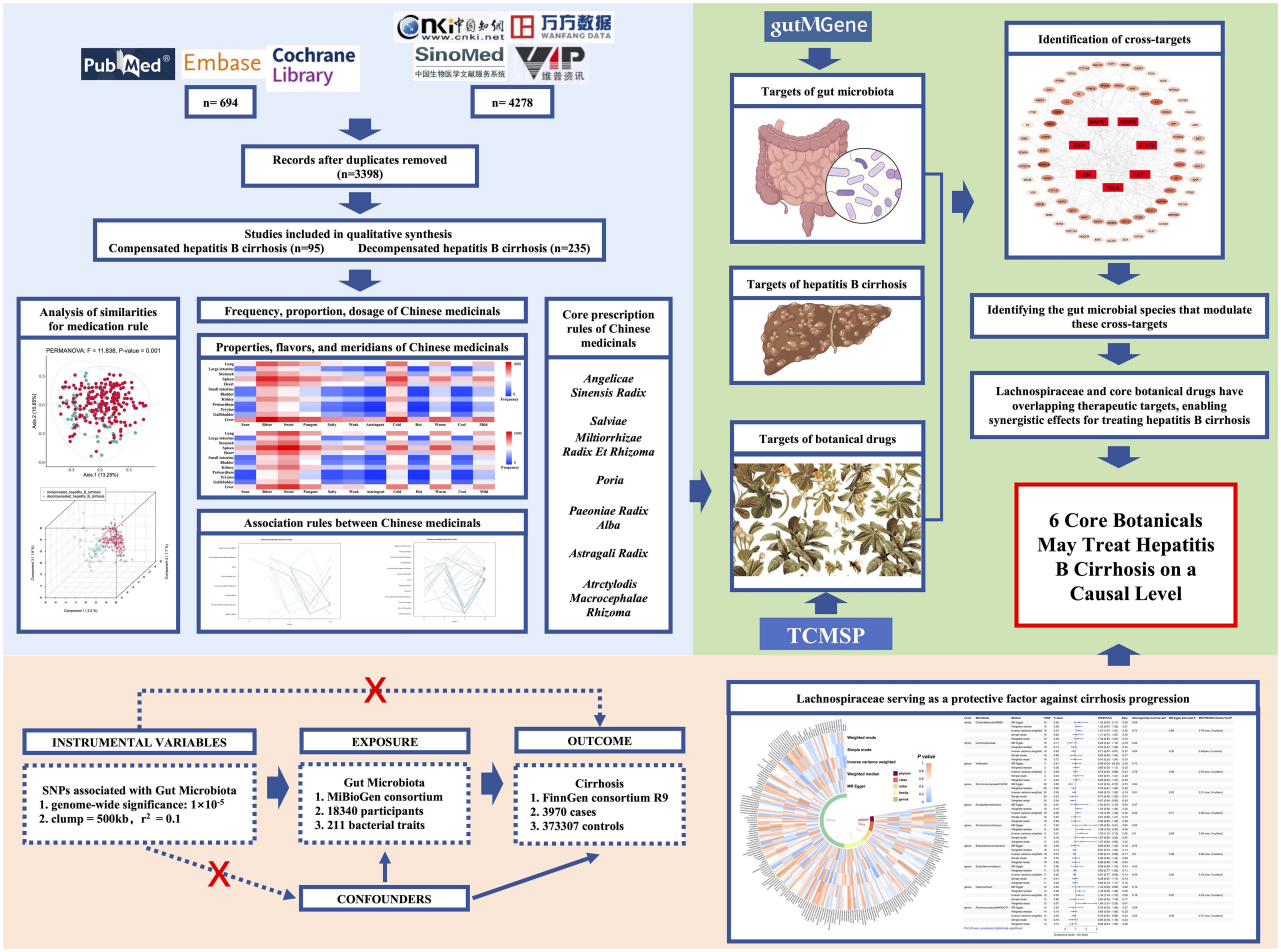
Overall flow figure of this study.

The general flow of this study is shown in **Figure 1**. Data source: Chinese National Knowledge Infrastructure (CNKI), Wanfang database (Wanfang), VIP database (VIP), Chinese Biomedical Literature database (CBM), PubMed, Embase and Cochrane Library databases were searched for clinical research literature on TCM for the treatment of hepatitis B cirrhosis from the time of database creation to May 2022. The search terms were (“Hepatitis B cirrhosis” OR “HBV-related hepatic cirrhosis” OR “HBV-liver cirrhosis “ OR “hepatitis B-caused cirrhosis” OR “cirrhosis” OR “liver cirrhosis”) AND (“traditional Chinese medicine” OR “Chinese medicine” OR “oriental medicine” OR “herbal medicine” OR “herb” OR “plant” OR “prescription” OR “decoction” OR “natural product” OR “Chinese herbal formula” OR “Chinese herbal medicine” OR “ Chinese medicinals”). We have provided detailed search strategies and the details of original literatures in **Additional File 1: S1**. All relevant literature in the database was screened using Endnote to obtain the relevant articles.

### Inclusion and exclusion criteria

Inclusion criteria: (1) The included studies were randomized controlled trials or observational clinical studies that enrolled at least one group of subjects receiving TCM treatment. (2) The study subjects were all patients diagnosed with hepatitis B cirrhosis according to the diagnostic criteria outlined in China’s 2019 guidelines for the prevention and treatment of chronic hepatitis B. The criteria include a clear history of HBV infection and evidence of cirrhosis based on pathological or imaging examination. (3) The interventions included herbal soup, herbal granules and other internal Chinese medicines, all of which had specified dosages. (4) In case of duplicate publications, the literature with the most complete clinical information was selected for inclusion. Exclusion criteria: (1) Case reports, reviews, conference reports, animal experiments and theoretical discussion articles. (2) TCM treatment that included external therapies such as acupuncture. (3) Patients included in the literature with comorbid cirrhosis due to other etiologies, such as hepatitis C, hepatitis D, and non-alcoholic fatty liver disease. (4) Literature in which it cannot be determined whether the patient has compensated or decompensated cirrhosis.

### Data extraction and normalization

Two researchers (ZC and YP) independently searched databases and screened relevant literature. WJJ discussed any disagreements or made further decisions. Information regarding title, author, publication date, prescription composition, and dosage were recorded for each article. The names of Chinese medicinals were standardized in accordance with the Pharmacopoeia of the People’s Republic of China (2020 edition).

### Screening of active ingredients and cross-targets

We obtained the bioinformatics data for 6 core botanical drugs from the TCMSP database (http://tcmspw.com/tcmsp.php). According to the ADME parameters, the active ingredients and related corresponding targets were screened based on the oral bioavailability (OB) screening threshold ≥ 30% and drug-like properties (DL) ≥ 0.18 (Lucas et al., 2019). In the case of compounds without corresponding targets, the SMILES structures of the compounds were downloaded from the PubChem (https://pubchem.Ncbi.nlm.nih.gov/) and uploaded to the SwissTargetPrediction database (http://www.swisstargetprediction.ch/) for target prediction (Gfeller et al., 2014). The targets of compound were entered into the Uniprot database (https://www.uniprot.org), normalizing the names of the genes corresponding to the target proteins. Additionally, we conducted a literature search to identify any targets associated with these 6 botanicals that were not present in the database. The GeneCards database (https://www.genecards.org), DisGeNET database (https://www.disgenet.org/) were searched for disease targets known to be associated with hepatitis B cirrhosis (Fishilevich et al., 2016; Piñero et al., 2021). Access drug targets recommended for the treatment of hepatitis B cirrhosis in the AASLD 2018 Hepatitis B Guidelines in the Drug Bank database (https://go.drugbank.com/) (Terrault et al., 2018; Wishart et al., 2018). GutMGene (http://bio-annotation.cn/gutmgene/) was used to retrieve the metabolites of gut microbes. We utilized the Similarity Ensemble Approach (SEA) (https://sea.bkslab.org/) and SwissTargetPrediction (STP) (http://www.swisstargetprediction.ch/) to predict the targets of metabolites(Keiser et al., 2007; Gfeller et al., 2014).

### Construction of gut microbiota action network based on core targets

Targets closely associated with cirrhosis were considered to overlapping targets of intestinal microbiota metabolites, disease, and botanical drugs. The String database (https://string-db.org/) was used to predict the interactions between targets. We utilized the Cytohubba plugin in Cytoscape 3.9.0 software to visualize interactions and identify the core targets. Based on the Kyoto Encyclopedia of Genes and Genomes (KEGG) and the connection between gut microbiota, metabolites, as provided by the GutMGene Database, we constructed an interaction network involving gut microbiota, metabolites, core targets, and signaling pathways.

### Selection of data sources for Mendelian randomization

We obtained genome-wide association data for the gut microbiota from the MiBioGen (https://mibiogen.gcc.rug.nl), which included 18,340 individuals. The dataset contained 122,110 host genetic variants mapped to genetic loci associated with abundance levels in 211 taxonomic groups, including 9 phyla, 16 classes, 20 orders, 35 families, and 131 genera (Kurilshikov et al., 2021). Cirrhosis data were obtained from the FinnGen consortium R9 release, which included 3970 cirrhosis cases and 373,307 controls (Emdin et al., 2020). Information on genotypes, cohorts, and other data from the FinnGen consortium is available on their website (https://www.finngen.fi/fi).

### Mendelian randomization analysis

#### Three hypotheses

To fulfill the three key assumptions of MR, we followed the approach below. Firstly, we selected exposure data with *P* < 1 × 10^-5^ to obtain more correlation results, since few GWAS-identified gut microbiota loci reached *P* < 1 × 10 ^-8^ (Liu et al., 2022). We set clump = 500 kb and r^2 = ^0.1 to exclude chained unbalanced SNPs. Moreover, we calculated the F-statistic for each SNP and only retained strongly correlated (F-statistic > 10) instrumental variables(Burgess and Thompson, 2011). Secondly, we used PhenoScanner V2 (http://www.phenoscanner.medschl.cam.ac.uk/) to assess genetic variation for confounding factors (including hepatitis A, hepatitis C, alcoholism, and autoimmune hepatitis) to prevent potential pleiotropy (Staley et al., 2016). Thirdly, we conducted a reverse MR analysis through the mr_steiger function to verify whether genetic variants affected the results only through exposure and whether exposure had a directional causal effect on the results (Hemani et al., 2017).

### Sensitivity and horizontal multiplicity analysis

We used random effects inverse variance weighting (IVW) as the primary analysis since it is the most robust method that can control for multiple biases (e.g., independence of genetic variants, balance, validity) through weighted averaging and has higher statistical efficacy when sample sizes are large (Burgess et al., 2013; Burgess et al., 2017). MR-PRESSO and MR-Egger assessed potential horizontal pleiotropy (Bowden et al., 2015; Verbanck et al., 2018). MR-PRESSO can detect potential outliers, correct for horizontal pleiotropy, and maintain the integrity of the original data, while MR-Egger can account for other biases (such as measurement error and genetic heterogeneity) to improve the accuracy and reliability of causal effects. Moreover, both MR-PRESSO and MR-Egger can perform sensitivity analyses to assess the reliability and stability of causal effects by testing the impact of outliers on the results. Additionally, Cochran’s Q-test assessed heterogeneity among genetic instruments as a reliable nonparametric method.

### Data analysis

All statistical analyses were done with R studio (version 4.1.3). Graphing or data analysis was performed using arules (version 1.7-3), ggplot2 (version 3.4.2), TwosampleMR (version 0.5.7), MR-PRESSO (version 1.0).

## Results

### Analysis of similarities for medication rules

**Figure 2:**
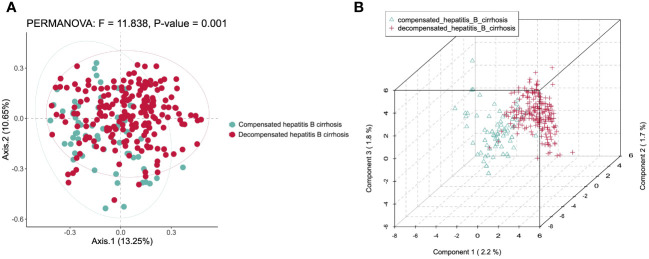
Analysis of similarities for medication rules. **(A)** Bray-Curtis PCoA. **(B)** PLS-DA.

By Bray-Curtis PCoA of Chinese medicinals in clinical trials to carry out diversity clustering, which appeared strong aggregation. In PCoA analysis, PC1 explained 13.25% and PC2 explained 10.65% of the total variation. The result of permanova revealed a strong F value of 11.838, and *P* < 0.01 **Figure 2A**. It indicates that the core Chinese medicinals are with big variations in different stages of hepatitis B cirrhosis. It is meaningful to analyze the medication rules by dividing cirrhosis into compensated and decompensated stages. We verified the results with PLS-DA, component 1 explained 2.2%, component 2 explained 1.7% and component 3 explained 1.8% of the total variation. The 3D PLS-DA model also demonstrated clear separation and tight clustering by disease stage **Figure 2B**.

### Frequency, dosage, and efficacy of Chinese medicinals

#### Compensated hepatitis B cirrhosis

A total of 95 studies involving 98 prescriptions were included. One prescription contained a maximum of 20 Chinese medicinals and a minimum of 6, with an average of 10.97 per prescription. There were 12 Chinese medicinals that were used more than 20 times (with 511 times), accounting for 47.53% of the total frequency **Table 1**. The 6 Chinese medicinals with the highest frequency of prescription were *Paeoniae Radix Alba* (58 times, 5.40% of total Chinese medicinals, 59.18% of the total prescriptions); *Salviae Miltiorrhizae Radix Et Rhizoma* (54 times, 5.02%, 55.10%); *Bupleuri Radix* (54 times, 5.02%, 55.10%); *Trionycis Carapax* (53 times, 4.93%, 54.08%); A*trctylodis Macrocephalae Rhizoma* (53 times, 4.93%, 54.08%); *Poria* (49 times, 4.56%, 50.00%). It is worth noting that the frequency of Chinese medicinals for activating blood cir-culation to dissipate blood stasis, dispersing stagnated hepatoqi and spleen-invigorating amounted to 985 times. The dosages of *Paeoniae Radix Alba, Salviae Miltiorrhizae Radix Et Rhizoma, Bupleuri Radix, Atrctylodis Macrocephalae Rhizoma, Poria, Angelicae Sinensis Radix, Persicae Semen, Paeoniae Radix Rubra, and Curcumae Radix* exceeded the recommended dosages, indicating that the administration of this category of Chinese medicinals is important in the treatment of compensated hepatitis B cirrhosis.

**Table 1:** Chinese medicinals appearing over 20 times in prescriptions of compensated hepatitis B cirrhosis.

Chinese medicinals	Frequency	Rate	Proportion	Minimum dosage	Maximum dosage	Average dosage	Recommended dosage
Paeonia lactiflora Pall. [Paeoniaceae; *Paeoniae Radix Alba*]	58	59.18%	5.40%	6	30	16.41 ± 5.99	6-15g
Salvia miltiorrhiza Bunge. [Lamiaceae; *Salviae Miltiorrhizae Radix Et Rhizoma*]	54	55.10%	5.02%	6	30	17.30 ± 6.37	10-15g
Bupleurum chinense DC. [Apiaceae; *Bupleuri Radix*]	54	55.10%	5.02%	6	30	11.89 ± 3.64	3-10g
*Trionycis Carapax*	53	54.08%	4.93%	4	30	16.91 ± 6.19	9-24g
Atractylodes macrocephala Koidz. [Asteraceae; *Atrctylodis Macrocephalae Rhizoma*]	53	54.08%	4.93%	5	30	13.17 ± 5.42	6-12g
Poria cocos (Schw.) Wolf. [Polypores; *Poria*]	49	50.00%	4.56%	10	32	18.29 ± 7.18	10-15g
Astragalus mongholicus Bunge [Fabaceae; *Astragali Radix*]	43	43.88%	4.00%	10	50	23.86 ± 7.91	9-30g
Angelica sinensis (Oliv.) Diels [Apiaceae; *Angelicae Sinensis Radix*]	40	40.82%	3.72%	6	30	13.2 ± 5.92	6-12g
Glycyrrhiza glabra L. [Fabaceae; *Glycyrrhizae Radix Et Rhizoma*]	37	37.76%	3.44%	3	15	6.73 ± 2.39	2-10g
Prunus persica (L.) Batsch [Rosaceae; *Persicae Semen*]	25	25.51%	2.33%	6	15	10.44 ± 1.92	5-10g
Paeonia lactiflora Pall. [Paeoniaceae; *Paeoniae Radix Rubra*]	23	23.47%	2.14%	8	30	16.43 ± 4.71	6-12g
Curcuma longa L. [Zingiberaceae; *Curcumae Radix*]	22	22.45%	2.05%	10	15	12.14 ± 2.85	3-10g

#### Decompensated hepatitis B cirrhosis

A total of 235 studies involving 241 prescriptions were included. A prescription contained a maximum of 24 Chinese medicinals and a minimum of 2, with an average of 12.27 per prescription. Of the 241 prescriptions, 204 Chinese medicinals appeared 2958 times. Among them, there were 20 Chinese medicinals used more than 40 times, which were used 1702 times, accounting for 57.54% of the total frequency **Table 2**. The most frequently Chinese medicinal was *Poria*, which appeared 173 times (71.78%). The following are most commonly used in descending order: *Poria, Astragali Radix, Atractylodis Macrocephalae Rhizoma, Salviae Miltiorrhizae Radix Et Rhizoma, Trionycis Carapax, Alismatis Rhizoma, Glycyrrhizae Radix Et Rhizoma*, etc. What is known is that these Chinese medicinals were preferred by those who have decompensated hepatitis B cirrhosis in TCM treatment. The high-frequency Chinese medicinals have a complicated range of actions for activating blood cir-culation to dissipate blood stasis, spleen-invigorating and diuresis-promoting. *Poria, Salviae Miltiorrhizae Radix Et Rhizoma, Atrctylodis Macrocephalae Rhizoma, Paeoniae Radix Rubra, Angelicae Sinensis Radix, Persicae Semen, Curcumae Radix, Alismatis Rhizoma, Polyporus, Arecae Pericarpium, Lycopi Herba, Plantaginis Semen, Artemisiae Scopariae Herba* were exceeding the recommended dosage. In addition, we found that the dosage tended to be higher in both compensated and decompensated hepatitis B cirrhosis stages, potentially due to the need for more active drug ingredients to achieve therapeutic effects in this end-stage liver disease.

**Table 2:** Chinese medicinals appearing over 40 times in prescriptions of decompensated hepatitis B cirrhosis.

Chinese medicinals	Frequency	Rate	Proportion	Minimum dosage	Maximum dosage	Average dosage	Recommended dosage
Poria cocos (Schw.) Wolf. [Polypores; *Poria*]	173	71.78%	5.85%	8	120	20.72 ± 15.03	10-15g
Astragalus mongholicus Bunge [Fabaceae; *Astragali Radix*]	166	68.88%	5.61%	10	60	27.55 ± 9.54	9-30g
Atractylodes macrocephala Koidz. [Asteraceae; *Atrctylodis Macrocephalae Rhizoma*]	163	67.63%	5.51%	6	40	16.58 ± 6.43	6-12g
Salvia miltiorrhiza Bunge. [Lamiaceae; *Salviae Miltiorrhizae Radix Et Rhizoma*]	122	50.62%	4.12%	10	40	20.78 ± 7.15	10-15g
*Trionycis Carapax*	107	44.40%	3.62%	8	30	19.29 ± 6.89	9-24g
Alisma plantago-aquatica subsp. orientale (Sam.) Sam. [*Alismataceae; Alismatis Rhizoma*]	107	44.40%	3.62%	6	30	16.35 ± 5.72	6-10g
Glycyrrhiza glabra L. [Fabaceae; *Glycyrrhizae Radix Et Rhizoma*]	95	39.42%	3.21%	3	20	7.55 ± 3.34	2-10g
Angelica sinensis (Oliv.) Diels [Apiaceae; *Angelicae Sinensis Radix*]	84	34.85%	2.84%	8	25	13.55 ± 3.69	6-12g
Bupleurum chinense DC. [Apiaceae; *Bupleuri Radix*]	78	32.37%	2.64%	5	30	11.36 ± 3.31	3-10g
Polyporus umbellatus (Pers.) Fries [Polyporaceae; *Polyporus*]	78	32.37%	2.64%	10	50	17.83 ± 7.63	6-12g
Areca catechu L. [Arecaceae; *Arecae Pericarpium*]	73	30.29%	2.47%	10	30	17.92 ± 6.11	5-10g
Paeonia lactiflora Pall. [Paeoniaceae; *Paeoniae Radix Rubra*]	68	28.22%	2.30%	5	40	14.17 ± 6.94	6-12g
Codonopsis pilosula (Franch.) Nannf. [Campanulaceae; *Codonopsis Radix*]	60	24.90%	2.03%	10	30	18.15 ± 6.11	9-30g
Lycopus lucidus Turcz. ex Benth var. hirtus Regel [Labiatae; *Lycopi Herba*]	53	21.99%	1.79%	8	30	18.38 ± 7.61	6-12g
Prunus persica (L.) Batsch [Rosaceae; *Persicae Semen*]	52	21.58%	1.76%	6	20	11.10 ± 2.26	5-10g
Paeonia lactiflora Pall. [Paeoniaceae; Paeoniae Radix Alba]	51	21.16%	1.72%	10	30	14.94 ± 5.06	6-15g
Plantago asiatica L. [Plantaginaceae; *Plantaginis Semen*]	44	18.26%	1.49%	8	30	16.84 ± 5.88	9-15g
Artemisia capillaris Thunb. [Asteraceae; *Artemisiae Scopariae Herba*]	45	18.67%	1.52%	9	60	21.29 ± 11.89	6-15g
Curcuma longa L. [Zingiberaceae; *Curcumae Radix*]	44	18.26%	1.49%	10	20	14.23 ± 3.72	3-10g
Citrus × aurantium L. [Rutaceae; *Citri Reticulatae Pericarpium*]	40	16.60%	1.35%	5	25	11.28 ± 3.50	3-10g
Codonopsis pilosula (Franch.) Nannf. [Campanulaceae; C*odonopsis Radix*]	173	71.78%	5.85%	8	120	20.72 ± 15.03	10-15g

### Properties, flavors, and meridians of Chinese medicinals

#### Compensated hepatitis B cirrhosis

**Figure 3:**
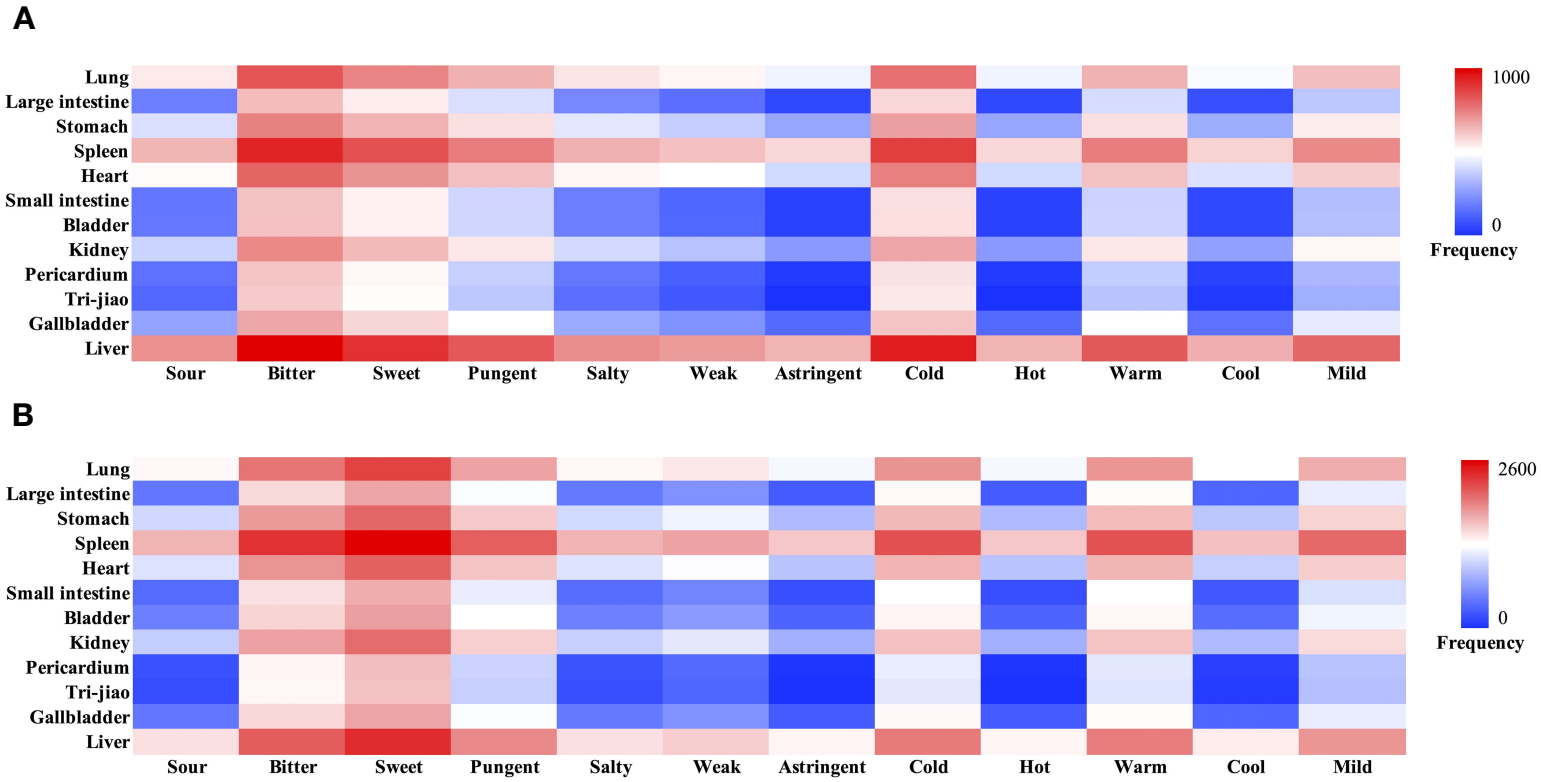
Heatmap of Properties, flavors and meridians of hepatitis B cirrhosis. **(A)** Compensated hepatitis B cirrhosis. **(B)** Decompensated hepatitis B cirrhosis.

According to the 2020 edition of the Chinese Pharmacopoeia, we analyzed the 34 most frequently used Chinese medicinals in compensated hepatitis B cirrhosis in terms of properties, flavors, and meridians **Additional File 1: S2**. *Herba Hedyotis* is not included in the 2020 edition of the Chinese Pharmacopoeia, the information on the properties, flavors, and meridians is obtained from the Chinese Materia Medica. The results showed that the cumulative frequency of flavors was recorded 1251 times, astringent Chinese medicinals were not recorded, and the highest frequency of use was for the bitter (451 times, 37.87%), sweet (334 times, 28.04%), and pungent (225 times, 18.89%). The cumulative frequency of the properties was recorded 803 times, hot Chinese medicinals were not recorded, and the highest frequency of use was for the cold (381 times, 47.45%), warm (221 times, 27.52%) and mild (190 times, 23.66%). A total of 11 meridian attributions were involved in the analysis of 34 Chinese medicinals, with a cumulative frequency of 1987 times. A total of 11 meridian attributions were involved in the analysis of 34 Chinese medicinals, with a cumulative frequency of 1987 times. The highest frequency of meridians was Liver Meridian of Foot-Jueyin (507 times, 25.52%), the Spleen Meridian of Foot-Taiyin (419 times, 21.09%), and Lung Meridian of Hand-Taiyin (290 times, 14.59%) **Figure 3A**.

#### Decompensated hepatitis B cirrhosis

According to the 2020 edition of the Chinese Pharmacopoeia, we analyzed the 41 high-frequency Chinese medicinals used more than 20 times in decompensated hepatitis B cirrhosis in terms of properties, flavors, and meridians **Additional File 1: S3**. *Herba Hedyotis* is not included in the 2020 edition of the Chinese Pharmacopoeia, the information on the properties, flavors, and meridians is obtained from the Chinese Materia Medica. The results showed that the cumulative frequency of flavors was recorded 3377 times, astringent Chinese medicinals were not recorded, and the highest frequency of use was for the sweet (1286 times, 38.08%), bitter (956 times, 28.31%), and pungent (659 times, 19.51%). The cumulative frequency of the properties was recorded 2146 times, hot Chinese medicinals were not recorded, and the highest frequency of use was for the cold (760 times, 35.41%), warm (746 times, 34.76%) and mild (596 times, 27.77%). The 41 Chinese medicinals involved all the meridians, with a cumulative frequency of 5964 times, and the highest frequency of use was in the order of Spleen Meridian of Foot-Taiyin (1292 times, 21.66%), Liver Meridian of Foot-Jueyin (1009 times, 16.92%), and Lung Meridian of Hand-Taiyin (861 times, 14.44%) **Figure 3B**. There are considerable variations in the treatment of compensated and decompensated hepatitis B cirrhosis, as evidenced by the noticeably increased frequency of sweet-warm Chinese medicinals and the decrease of bitter-cold Chinese medicinals in decompensated hepatitis B cirrhosis.

### Association analysis between Chinese medicinals

#### Compensated hepatitis B cirrhosis

**Figure 4:**
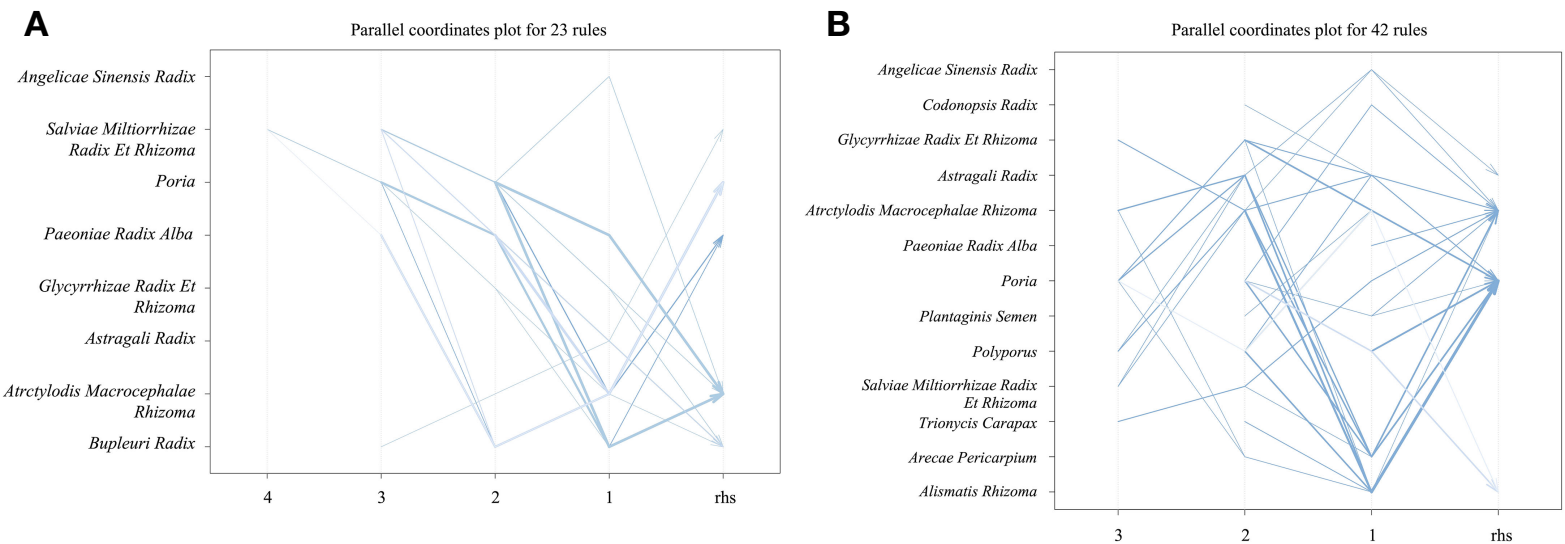
Parallel coordinates plot of hepatitis B cirrhosis. Each line indicates a combination. The depth of the color of the line indicates the degree of lift, the darker the blue color, the higher the degree of lift. The width of the line indicates the degree of confidence, the wider the line, the higher the degree of confidence. **(A)** Compensated hepatitis B cirrhosis. **(B)** Decompensated hepatitis B cirrhosis.

To clarify the clinical application of Chinese medicinals in combination, we performed association analysis of high-frequency Chinese medicinals (frequency >10 times) using Apriori in R-Studio. After the minimum support was set to 0.16 and the minimum confidence was set to 0.9, a total of 23 association rules appeared, and all association rules had a lift value greater than 1, indicating that all association rules were valid **Additional File 1: S4**. Among the 23 association rules, *{Bupleuri Radix, Poria}=>{Atrctylodis Macrocephalae Rhizoma}*, *{Paeoniae Radix Alba, Poria}=>{Atrctylodis Macrocephalae Rhizoma}, {Atrctylodis Macrocephalae Rhizoma, Paeoniae Radix Alba}=>{Poria}* had the highest support, all with 34.69%.{*Astragali Radix, Paeoniae Radix Alba, Salviae Miltiorrhizae Radix Et Rhizoma}=>{Bupleuri Radix}* had the highest confidence level at 100%. *{Astragali Radix, Paeoniae Radix Alba, Salviae Miltiorrhizae Radix Et Rhizoma} => {Bupleuri Radix}* had the highest degree of lift at 1.81. We found 8 botanical drugs under the association rule: *Angelicae Sinensis Radix, Salviae Miltiorrhizae Radix Et Rhizoma, Poria, Paeoniae Radix Alba, Glycyrrhizae Radix Et Rhizoma, Astragali Radix, Atrctylodis Macrocephalae Rhizoma, Bupleuri Radix. Paeoniae Radix Alba, Atrctylodis Macrocephalae Rhizoma, Poria, Bupleuri Radix, Salviae Miltiorrhizae Radix Et Rhizoma* were identified as hubs in the parallel coordinates plot by association rules **Figure 4A**.

#### Decompensated hepatitis B cirrhosis

We conducted an association analysis of high-frequency Chinese medicinals (frequency >20 times) in decompensated hepatitis B cirrhosis. Similarly, the minimum support was set to 0.16 and the minimum confidence was set to 0.9. A total of 42 association rules appeared, all association rules had a lift value greater than 1, indicating that all association rules were valid **Additional File 1: S5**. Among the 42 association rules, *{Alismatis Rhizoma}=>{Poria}* had the highest support, all with 41.08%.*{Plantaginis Semen}=>{Atrctylodis Macrocephalae Rhizoma}, {Plantaginis Semen, Poria}=>{Atrctylodis Macrocephalae Rhizoma}, {Astragali Radix, Atrctylodis Macrocephalae Rhizoma, Polyporus}=>{Poria}* had the highest confidence level at 100%. *{Atrctylodis Macrocephalae Rhizoma, Polyporus, Poria}=>{Alismatis Rhizoma}* had the highest degree of lift at 2.09. By showing commonalities and general rules in association analysis, a total of 13 Chinese medicinals were emphasized: *Angelicae Sinensis Radix, Salviae Miltiorrhizae Radix Et Rhizoma, Poria, Paeoniae Radix Alba, Glycyrrhizae Radix Et Rhizoma, Astragali Radix, Atrctylodis Macrocephalae Rhizoma, Polyporus, Plantaginis Semen, Alismatis Rhizoma, Trionycis Carapax, Arecae Pericarpium, Codonopsis Radix. Atrctylodis Macrocephalae Rhizoma, Poria, Polyporus, Alismatis Rhizoma, Astragali Radix, Arecae Pericarpium* were identified as hubs in the parallel coordinates plot **Figure 4B**. *Angelicae Sinensis Radix, Salviae Miltiorrhizae Radix Et Rhizoma, Poria, Paeoniae Radix Alba, Glycyrrhizae Radix Et Rhizoma, Astragali Radix, Atrctylodis Macrocephalae Rhizoma* were the core botanical drugs in the prescription, which are often used in combination. These botanical drugs may play an influential role in clinical treatment.

### Identification of targets for core botanicals, gut microbiota and hepatitis B cirrhosis

To further revealed the potential pharmacodynamic components, targets and related pathways in the treatment of hepatitis B cirrhosis, we analyzed 6 core botanical drugs by network analysis. We did not analyze the active ingredients and targets of *Glycyrrhizae Radix Et Rhizoma* since it was found in almost all TCM prescriptions and used in small amount. The active ingredients of *Angelicae Sinensis Radix, Salviae Miltiorrhizae Radix Et Rhizoma, Poria, Paeoniae Radix Alba, Astragali Radix, Atrctylodis Macrocephalae Rhizoma* were searched through literatures and TCMSP database (http://tcmspw.com/tcmsp.php), which screened based on OB≥30% and DL≥0.18 (Ru et al., 2014). We obtained information on the main components of 102 botanicals by conducting TCMSP database and the literature search **Additional File 1: S6**. Targets of active ingredient were retrieved from the TCMSP database, while missing active ingredient targets were predicted by SwissTargetPrediction database (http://www.swisstargetprediction.ch/) (Gfeller et al., 2014). The UniProt database (http://www.uniprot.org/) was used to normalize the names of 283 target names. We identified a total of 208 metabolites of the gut microbiota through GutMGene (http://bio-annotation.cn/gutmgene/) and searched for metabolites’ targets through the SEA(https://sea.bkslab.org/) and STP (http://www.swisstargetprediction.ch/) databases. A total of 1256 targets were identified in the SEA database, while 947 targets were identified in the STP database, with 668 overlapping targets identified. These targets are believed to be important for the gut microbiota to regulate various biological processes. The GeneCards database (https://www.genecards.org) and DisGeNET database (https://www.disgenet.org) were searched for currently known disease targets associated with hepatitis B cirrhosis (Fishilevich et al., 2016; Piñero et al., 2021). Access drug targets recommended for the treatment of hepatitis B cirrhosis in the AASLD 2018 Hepatitis B Guidelines in the Drug Bank database (https://go.drugbank.com/) (Terrault et al., 2018; Wishart et al., 2018). A total of 2191 disease targets were obtained.

### Identification of cross-targets and related biological effects

**Figure 5:**
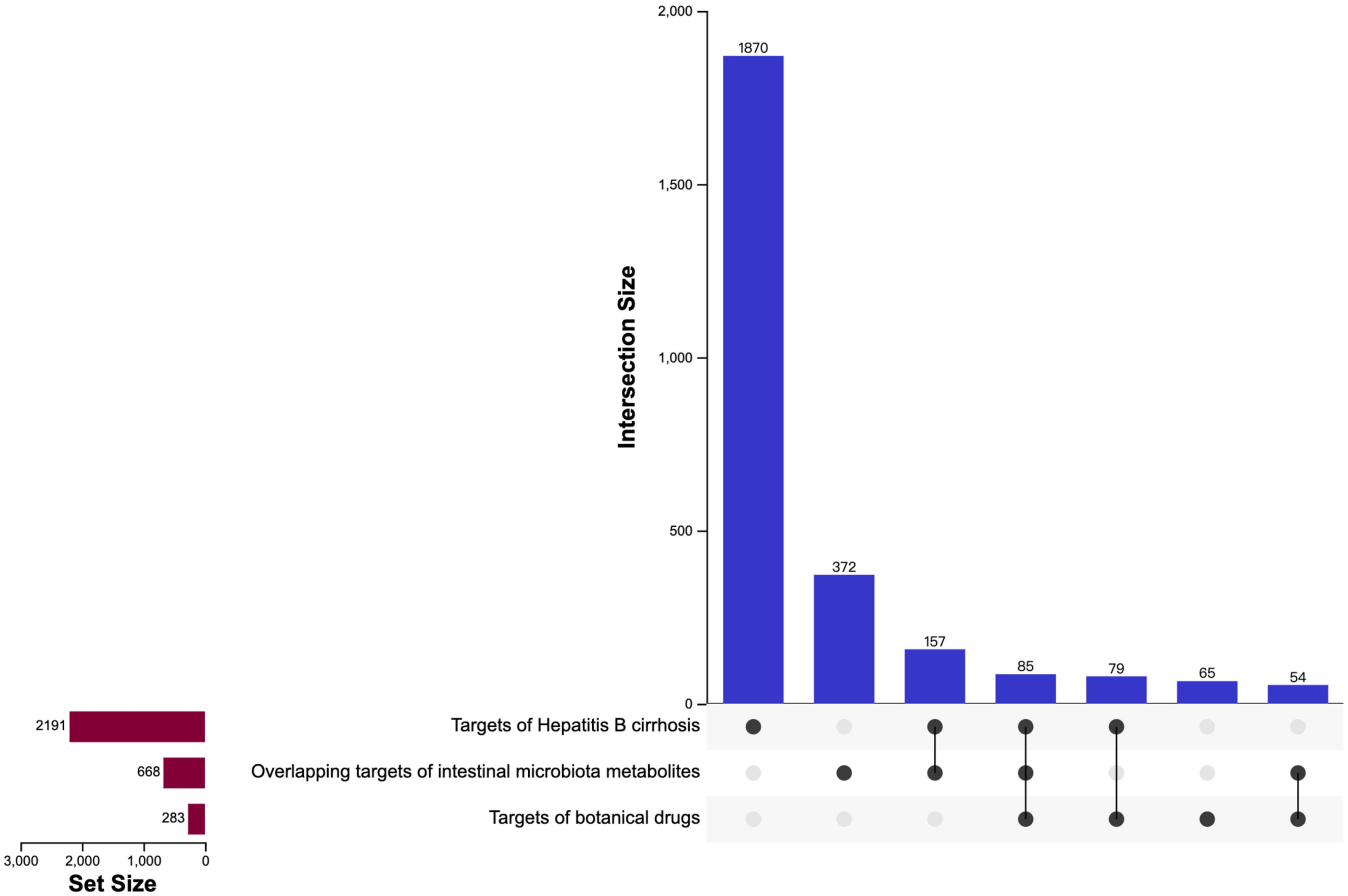
The UpSet plot of targets of hepatitis B cirrhosis, targets of botanical drugs and overlapping targets of intestinal microbiota metabolites. The isolated points are the unique targets in the project, and the connected points are the targets shared by 2 or 3 projects.

**Figure 6:**
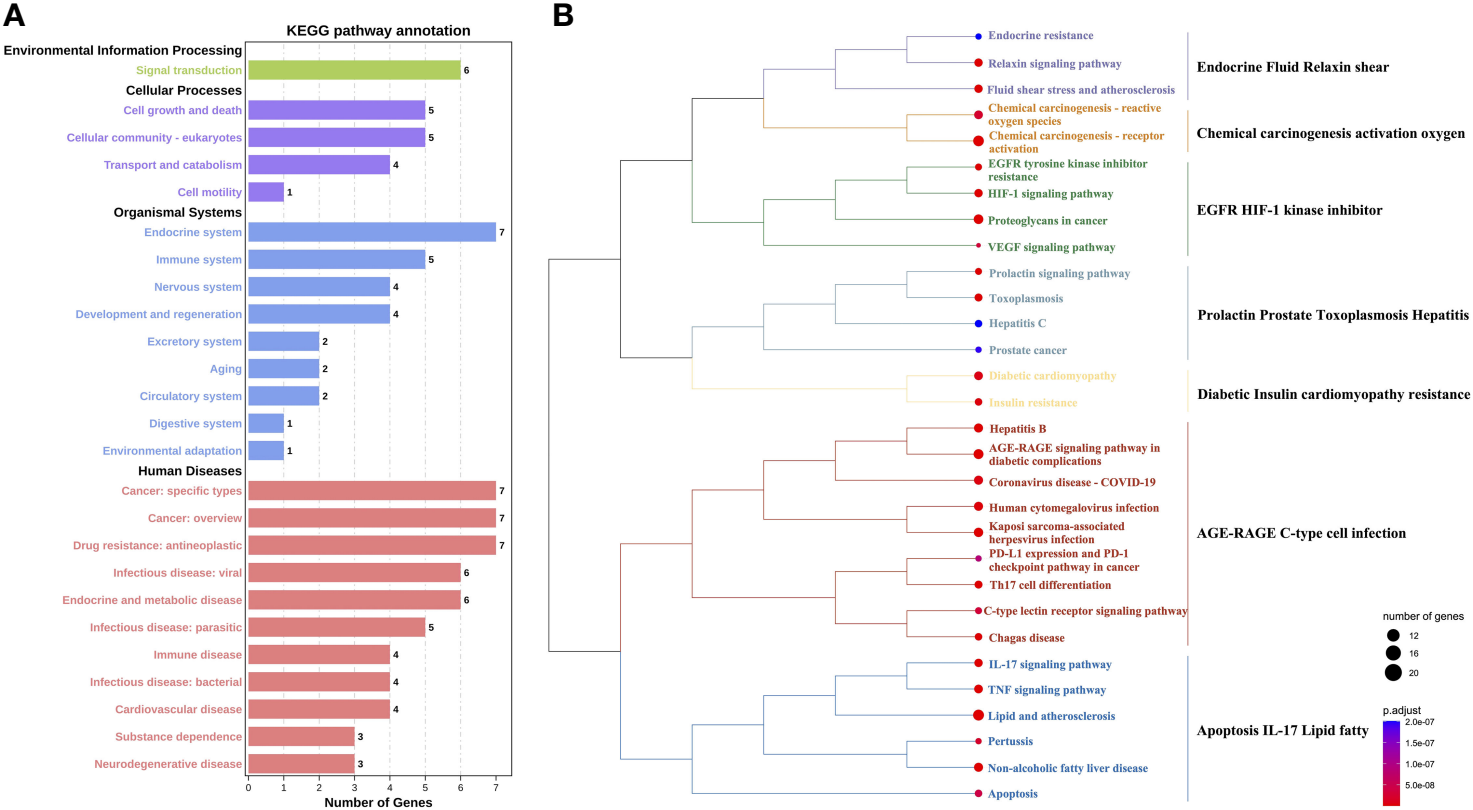
Analysis of the KEGG pathway for core targets. **(A)** KEGG pathway annotation. The x-axis is the number of genes. The y-axis is the pathway name. **(B)** Cluster Analysis of KEGG Pathway. On the left is the clustering tree. The node size indicates the number of targets, the larger the circle, the more targets are contained in the pathway. The color of the node indicates the importance of the pathway (smaller *P*-value is more important), darker red means smaller *P*-value and darker blue means larger *P*-value. On the right are the main roles of KEGG pathways under clustering, and pathways with uniform colors under the clustering tree have similar roles.

We conducted a Venn analysis on the targets of intestinal microbiota metabolites, the targets of botanical drugs, and the targets of hepatitis B cirrhosis. This analysis identified overlapping targets, which are considered important for the synergistic interaction of the six botanicals with the gut microbiota **Figure 5**; **Additional File 2: Table S1**. We obtained 85 cross-targets such as CYP3A4/CYP2C19/CYP1B1/CYP1A2/CYP1A1/CYP17A1, CASP8/CASP7/CASP3, MMP9/MMP3/MMP2/MMP1, which mainly belong to cytochrome P450 family, Caspase family, Matrix metalloproteinases family, etc. To further elucidate the biological functions of these targets, we performed KEGG pathway analysis on 85 cross-targets. KEGG pathway annotation clearly demonstrated the mechanisms of action of the botanicals **Figure 6**. The annotations of the pathways were divided into Environmental Information Processing, Cellular Processes, Organismal Systems and Human Diseases. The endocrine system and tumor-related disease pathways were significantly more enriched than other pathways, suggesting the botanicals may primarily act by modulating the internal environment to inhibit cirrhosis progression to malignancy.

### Construction of gut microbiota associations based on core targets

**Figure 7:**
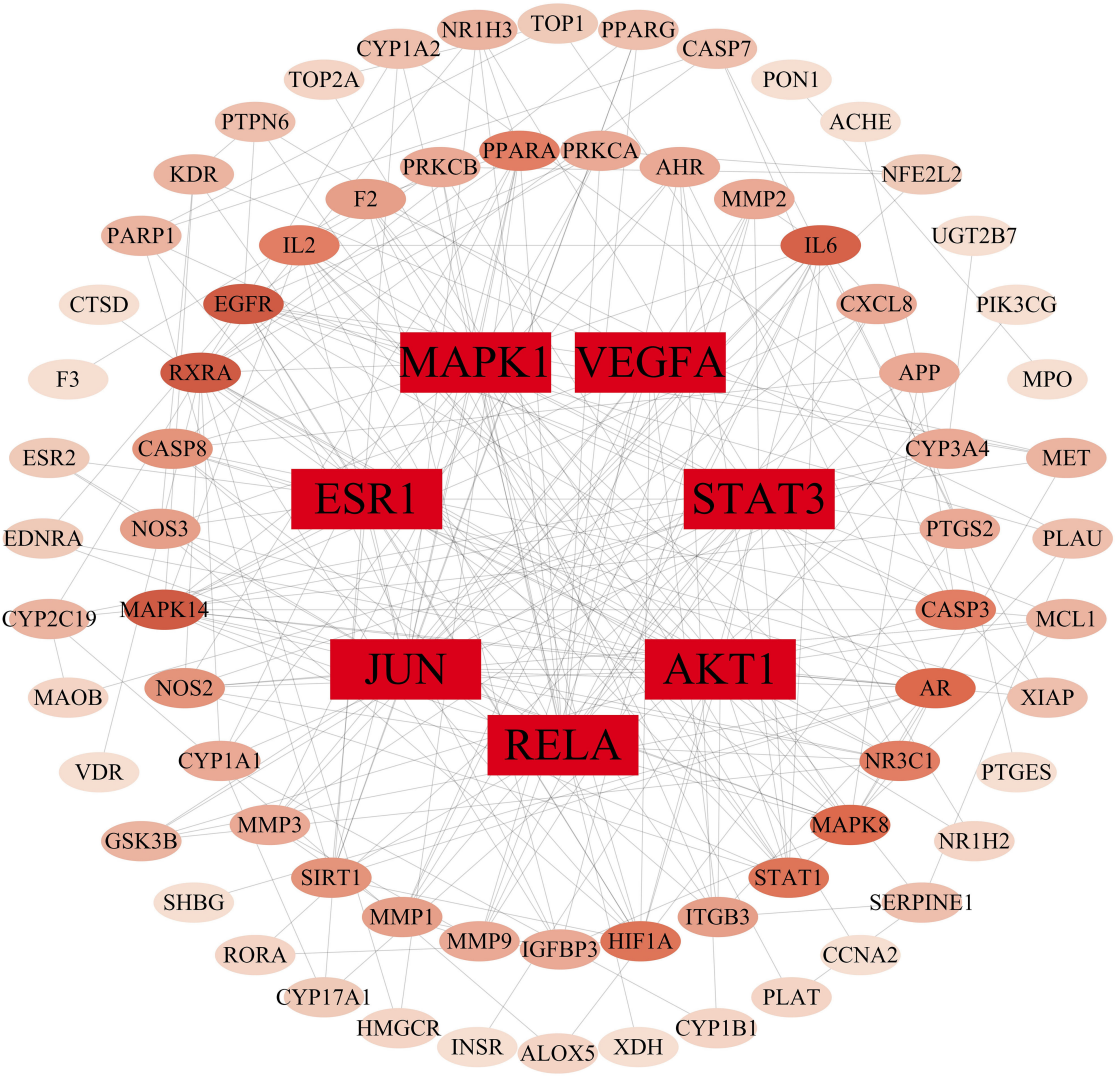
Protein interaction network plot for core targets. The depth of the color of the circle, the greater the probability of becoming a core target.

**Figure 8:**
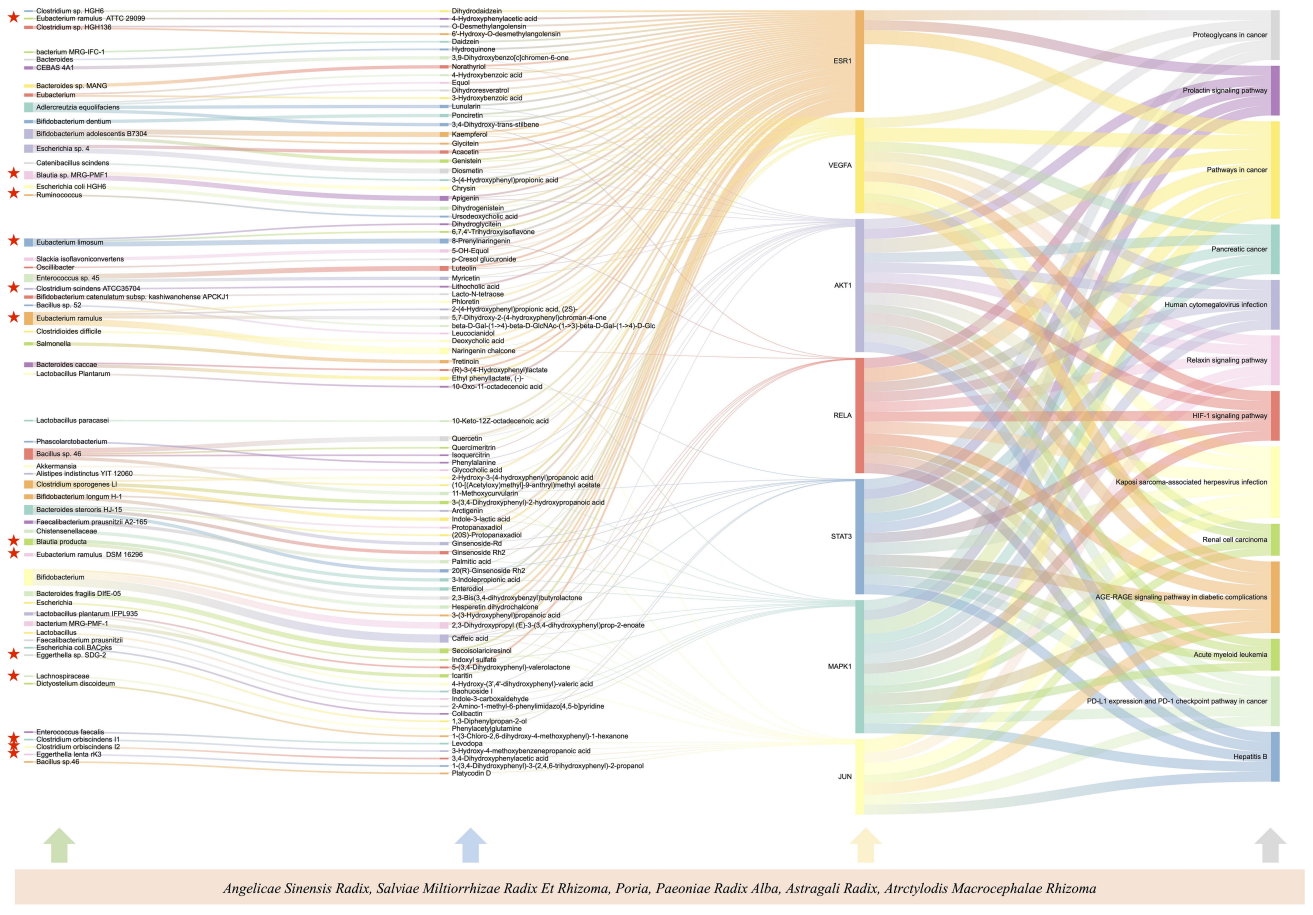
Sankey diagrams to represent the network relationships between gut microbiota, gut microbiota metabolites, targets, and pathways. The height of the square and the width of the line both represent the correlation with the target, the stronger the correlation, the taller the square and the wider the line. Gut microorganisms attributed to the Lachnospiraceae are highlighted.

By importing 85 cross-targets to the STRING database (https://string-db.org/), we predicted the interaction between targets. After the confidence level was set to 0.4, we imported the data into Cytoscape and used the degree value to explore the central targets in the network **Figure 7**. Node color intensity indicates more interactions and greater importance. The core proteins mainly include MAPK1, VEGFA, STAT3, AKT1, RELA, JUN and ESR1, which are the core targets of intervention in hepatitis B cirrhosis. This suggests targeted TCM drug development could incorporate these key botanicals to enhance therapeutic effects. Using the R Studio, we matched the 7 core targets with the gut microbiota and metabolites in the GutMgene database (http://bio-annotation.cn/gutmgene/) and established their network relationships with the gut microbiota, metabolites, and pathways. We constructed a Sankey diagram to represent the network relationships between gut microbiota, gut microbiota metabolites, targets, and pathways. **Figure 8**. In healthy symbiosis, gut microbiota maintain wellbeing by competing with foreign microbes and metabolizing beneficial substances like flavonoids. This mechanism becomes more critical in hepatitis B cirrhosis, as beneficial metabolites act on multiple targets through various pathways. Botanical drugs play a synergistic role in this process. First, they can serve as substrates to promote growth of beneficial microbiota. Additionally, botanical active ingredients resemble microbial metabolites or share therapeutic targets, allowing botanicals to mimic probiotic effects.

### Selection of Mendelian randomization instrumental variables

In the genetic analysis of bacterial taxa with cirrhosis, under the filter of removing linkage disequilibrium as well as the level of significance of loci (*P <*1×10^-5^), we obtained a total of 2934 SNPs, including 9 phyla (128 SNPs), 16 class (235 SNPs), 20 order (294 SNPs), 35 families (516 SNPs), and 131 genera (1761 SNPs). The F-values ranged from 14.59 to 88.43 and were all greater than 10 **Additional File 2: Table S2**.

### Associations of gut bacterial taxa with cirrhosis

**Figure 9:**
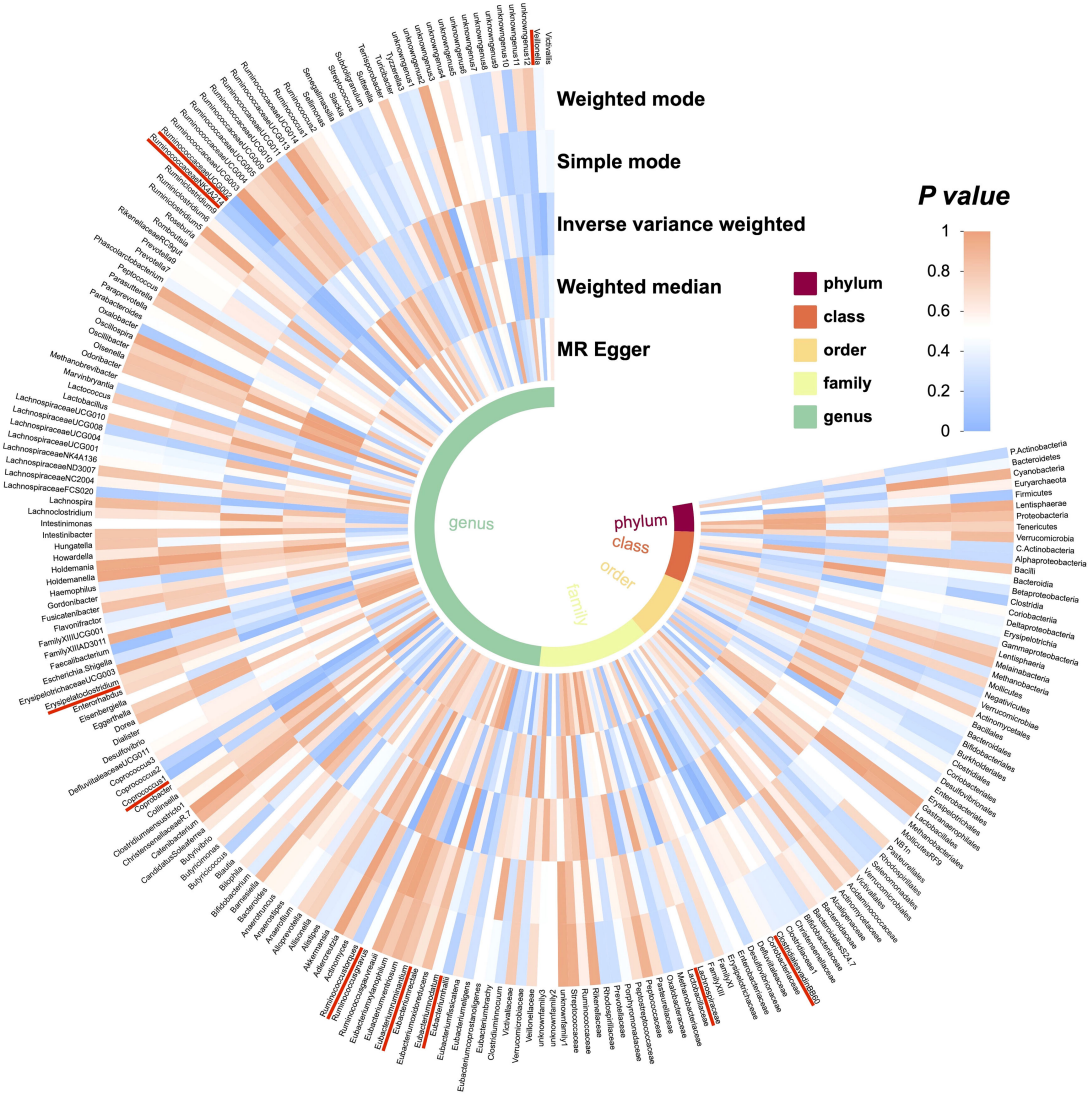
Preliminary MR analyses for the associations between gut microbiota and the risk of cirrhosis. The circle from the outer to the inner represented the weighted mode, simple mode, Inverse variance weighted, weighted median and MR-Egger, respectively. Gut microbiota was classified in order, phylum, class, family, and genus. The shades of color were reflections of the magnitude of the p-value as the label inside the circle.

**Figure 10:**
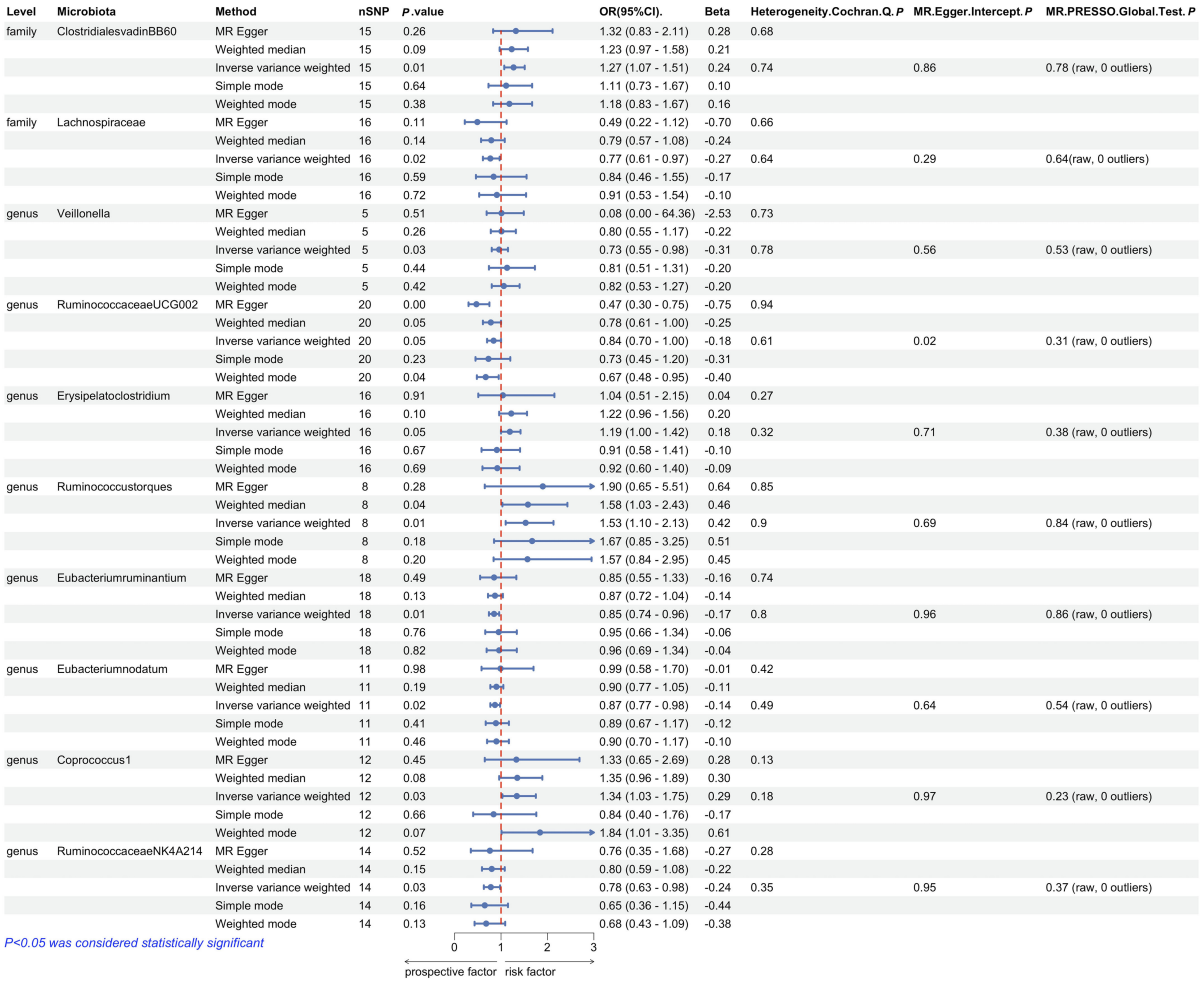
Forest plot of Mendelian randomization estimates between Gut microbiota and cirrhosis. The figure showed the MR-Egger, weighted median,nverse variance weighted, simple mode and weighted mode of significantly cirrhosis-associated gut microbiota taxa. The blue dots represent the estimates, and the blue bars represent the 95% confidence intervals of estimates. The OR > 1 indicates increased risk while< 1 indicates decreased risk. The last three columns are analyses of directional pleiotropy and heterogeneity; *P >*0.05 indicates no directional pleiotropy and heterogeneity.

We performed Mendelian randomization analysis mainly by five methods including Inverse variance weighted, MR-Egger, simple mode, weighted median, and weighted mode. The results of Mendelian randomization analysis for 211 taxa are shown in **Figure 9**. To query the associated diseases that put SNPs under the 10 gut microbiota taxa causally associated with cirrhosis, we used PhenoScanner V2 (http://www.phenoscanner.medschl.cam.ac.uk/). This tool allows us to search for genetic associations with various diseases and traits, including hepatitis A, hepatitis C, and alcohol consumption **Additional File 2: Table S3**. The absence of SNPs associated with confounding factors suggests that the causal relationship between gut microbiota and cirrhosis is not influenced by these factors. However, it is worth noting that Coprococcus1 and Erysipelatoclostridium had inconsistent beta value directions in the analysis results, which may indicate that different assumptions and constraints led to different results. In addition, we analyzed the study for heterogeneity and horizontal pleiotropy by MR-PRESSO as well as Cochran’s Q test. All *P*-values were greater than 0.05, indicating that there was no significant heterogeneity and horizontal pleiotropy **Figure 10**.

ClostridialesvadinBB60 (OR=1.27, 95%CI:1.07-1.51, *P*=0.006), Erysipelatoclostridium (OR=1.19, 95%CI:1.00-1.42, *P*=0.050), Ruminococcustorques (OR=1.53, 95%CI:1.10-2.13, P=0.012), and Coprococcus1 (OR=1.34, 95%CI:1.03-1.75, *P*=0.031) were determined to have a potentially positive causal effect on cirrhosis of Hepatitis B, thereby increasing Hepatitis B cirrhosis Risk. On the other hand, we found that 6 gut microbiota were associated with a reduced risk of hepatitis B cirrhosis, including Lachnospiraceae (OR=0.77, 95%CI:0.61-0.97, *P*=0.024), Veillonella (OR=0.73, 95%CI:0.55-0.98, *P*=0.03), RuminococcaceaeUCG002 (OR=0.84, 95%CI:0.70-1.00, *P*=0.049), Eubacteriumruminantium (OR=0.85, 95%CI:0.74-0.96, *P*=0.013), Eubacteriumnodatum (OR=0.87, 95%CI:0.77-0.98, *P*=0.024), RuminococcaceaeNK4A214 (OR=0.78, 95%CI: 0.63-0.98, *P*=0.034). These microbial communities may still be playing a therapeutic role after the onset of cirrhosis. After analyzing by the mr_steiger algorithm, we did not find any reverse causality of these 10 taxa on hepatitis B cirrhosis. Interestingly, Lachnospiraceae had a protective effect against hepatitis B cirrhosis, and according to our analysis above, the metabolites of Lachnospiraceae share common targets with the core phytopharmaceuticals. These key botanicals may treat hepatitis B cirrhosis by exerting similar biological effects as beneficial gut microbiota, with Mendelian randomization indicating such therapeutic effects occur through causal pathways. This elucidates the mechanism of action and the reason for widespread clinical use.

## Discussion

This study systematically summarized the results of previous studies on TCM for compensated/decompensated hepatitis B cirrhosis, which is the first time to analyze the TCM treatment of hepatitis B cirrhosis in stages. The core botanical drugs for the cirrhosis stage of hepatitis B were screened based on association rule analysis. Rather than demonstrate botanical target importance through animal or cell experiments, we utilized the GutMGene database to find microbiota sharing these targets. Mendelian randomization analysis verified the strong links between these microbes and hepatitis B cirrhosis. This illustrates the mechanism and significance of core phytomedicines in treating hepatitis B cirrhosis. This is the first systematic review of the prescription composition, dosing rules and treatment principles of TCM in the treatment of compensated/decompensated hepatitis B cirrhosis. Furthermore, this is the first study to combine network pharmacology with Mendelian randomization in analysis.

Data mining is the approach of searching for information with special relationships hidden in a large amount of data. The commonly used data mining approaches in the medical field include association rule analysis, cluster analysis, factor analysis, etc (Manhenke et al., 2013). Cluster analysis and factor analysis are known as exploratory classification methods, where similar data is scientifically grouped into smaller classes based on complex algorithms, and cannot figure out for core components. Association rule analysis has been applied in many aspects of TCM, including the relationships between diseases, symptoms and Chinese medicinals (Lu et al., 2020; Qi et al., 2021). The Apriori algorithm is mostly used to analyze the association rules, which can recognize the characteristics of combinations at the macro level, including searching for the core Chinese medicinals, finding the interconnection between Chinese medicinals and the overall medication rules.

Association rule analysis identified stage-specific prescriptions aligning with compensated and decompensated hepatitis B cirrhosis treatment principles. Since botanical drugs contain many active ingredients, incorrect application may lead to drug-induced liver injury (Ortega-Alonso and Andrade, 2018). Herb-induced liver injury (HILI) was formally recognized in 2011 as a major public health issue (Ma et al., 2023). Compared to Europe, herb use in Asia is more prevalent and the problem of HILI is more serious. Alarmingly, traditionally safe herbs increasingly cause HILI, potentially due to low quality, contaminated ingredients, or excessive dosage, as well as unsafe herb-herb interactions (irrational combinations of herbs) (Teschke et al., 2020). Currently, there is a global lack of widely applicable guidelines and regulations for proper, safe herbal medicine use. Therefore, reducing side effects and increasing efficacy are both aims of researching medication rules.

Upon data mining of Chinese medicinals, we found that the method of activating blood cir-culation to dissipate blood stasis is the common treatment for hepatitis B cirrhosis. Due to the theory of liver subjugating spleen, TCM treatment for hepatitis B cirrhosis often incorporates methods to strengthen the spleen to prevent the disease from worsening. The property, flavor, and meridian of Chinese medicinals are the accumulated experience of Chinese medicine for thousands of years in the treatment of diseases, which is another important information of Chinese medicinals (Chen et al., 2019). By analyzing the properties, flavors, and meridians, we found that there are some differences in Chinese medicinals between compensated and decompensated cirrhosis. Bitter, cold Chinese medicinals were more frequently used to treat compensated cirrhosis, and the meridian with the highest frequency is Liver Meridian of Foot-Jueyin, followed by Spleen Meridian of Foot-Taiyin, which is consistent with the principle of treatment. Sweet Chinese medicinals were commonly used in decompensated cirrhosis, and the frequency of cold did not differ much from that of warm, suggesting that decompensated cirrhosis requires a more involved approach to therapy. The pathological nature is intercalated with asthenia and sthenia, and the treatment should take into account multiple aspects, both to restore vital energy and eliminate pathogens. Spleen Meridian of Foot-Taiyin was the most frequent, followed by the Liver Meridian of Foot-Jueyin, with Chinese medicinals for spleen-invigorating increasing in frequency as the disease worsened. TCM is for the essence of the disease, focusing on the balance of yin and yang, treating the same disease with different methods is a specific example of the treatment means for hepatitis B cirrhosis, and the prevention of deterioration of existing diseases is an important principle of TCM treatment, the previous data mining for hepatitis B cirrhosis have ignored this point.

The second half of this study involves a fusion analysis of network pharmacology and Mendelian randomization. It describes the reasons for the frequent use of the core botanicals and the mechanisms by which they are used to treat cirrhosis of Hepatitis B. Previous studies (Li et al., 2022; Zhou et al., 2022) have utilized network pharmacology primarily to explore the potential mechanisms of drug action and demonstrate them through animal or cellular experiments. However, it is worth noting that some botanicals have numerous therapeutic targets for diseases but demonstrate limited clinical efficacy. Through analyzing the therapeutic targets, we identified gut microbiota possessing similar effects to the botanicals. Previous studies (Bajaj et al., 2018; Shu et al., 2022) have reported an association between the gut microbiota and cirrhosis, and our findings are consistent with the suggestion that Lachnospiraceae and Ruminococcaceae are protective factors against cirrhosis. Lachnospiraceae is the main producers of short-chain fatty acids (SCFA), including propionic, butyric, and acetic acids, which are produced by fermenting dietary cellulose and other complex polysaccharides (Vacca et al., 2020; Xie et al., 2022). SCFAs are important for gut health, as they provide energy to intestinal cells, maintain the integrity of the intestinal mucosal barrier, modulate the immune system, and reduce inflammation.

In conclusion, we found some variations in the medication rule between compensated and decompensated hepatitis B cirrhosis. *Angelicae Sinensis Radix, Salviae Miltiorrhizae Radix Et Rhizoma, Poria, Paeoniae Radix Alba, Astragali Radix, Atrctylodis Macrocephalae Rhizoma* may be botanical drugs for the treatment of hepatitis B cirrhosis. We have conducted a preliminary exploration of the therapeutic mechanisms of botanical drugs through network analysis and Mendelian randomization. This study on the pharmacological mechanisms of botanical drugs provides new ideas for new drug discovery and development.

This study has several strengths. Firstly, we identified the core botanicals currently used in the treatment of hepatitis B cirrhosis through data mining methods, which is important for optimizing the clinical regimen for patients. Secondly, we used MR analysis to establish a causal relationship between gut microbiota and hepatitis B cirrhosis, while accounting for confounding factors and reversing causal inference. Lastly, we obtained genetic variation in gut microbiota from the largest available GWAS meta-analysis, ensuring the validity of instrumental variables in MR analysis.

However, there are also some limitations to this study. Firstly, we were unable to determine if these botanicals produce more beneficial metabolites in the gut, and we did not investigate the combined effect of gut flora and botanicals. Secondly, Due to the limitations of GWAS data, the Mendelian randomization analysis was for a European population. Despite the issue of ethnographic differences, our results are consistent with previous studies in Asia, suggesting that the protective effects of these core microorganisms (e.g., Lachnospiraceae) may be consistent across populations. Thirdly, our study integrated and analyzed bioinformatic data from multiple sources, but lacked the necessary experimental validation. Future studies could validate the effects of these key botanicals on the gut microbiota of hepatitis B cirrhosis patients via clinical trials utilizing 16S sequencing alongside multi-omics analysis.

## Data availability statement

The datasets analyzed during the current study are available in MiBioGen repository (https://mibiogen.gcc.rug.nl/), FinnGen repository (https://www.finngen.fi/) and GutMgene repository (http://bio-annotation.cn/gutmgene/).

## Author contributions

CZ: Writing – original draft. JW: Conceptualization, Writing – original draft. PY: Conceptualization, Writing – review & editing. JY: Data curation, Writing – review & editing. TL: Software, Writing – review & editing. RJ: Writing – review & editing. SW: Data curation, Writing – review & editing. PS: Writing – review & editing. LY: Writing – review & editing. HX: Funding acquisition, Writing – review & editing.
