# Supplementary material for: Convergent application of traditional Chinese medicine and gut microbiota in ameliorate of cirrhosis: a data mining and Mendelian randomization study
Source: Front Cell Infect Microbiol. 2023 Nov 6;13:1273031. doi: 10.3389/fcimb.2023.1273031 (PMC10657829; doi:10.3389/fcimb.2023.1273031)
Supplement: Supplementary file 1 [file Table_1.docx]

**Supplementary Files S1-S6**

[Supplementary File S1. Search strategies for databases and the details of original literatures 1](#_Toc115345658)

[Supplementary File S2. High frequency herbs for compensated hepatitis B cirrhosis 6](#_Toc115345659)

[Supplementary File S3. High frequency herbs for decompensated hepatitis B cirrhosis 8](#_Toc115345660)

[Supplementary File S4. 23 association rules of compensated hepatitis B cirrhosis 10](#_Toc115345661)

[Supplementary File S5. 42 association rules of decompensated hepatitis B cirrhosis 12](#_Toc115345662)

[Supplementary File S6. 102 Active Ingredients of Herbs 15](#_Toc115345663)

**Supplementary File S1. Search strategies for databases and the details of original literatures**

**CNKI**

(SU='乙型肝炎肝硬化' + '乙型病毒性肝炎肝硬化' +'乙肝肝硬化' )

AND

(SU='中医' + '中药' + '中西医'+'中医药'+ '方剂' + '方药')

**Wanfang**

(乙型肝炎肝硬化OR乙型病毒性肝炎肝硬化OR乙肝肝硬化)

AND

(中医OR中药OR中西医OR中医药OR方剂OR方药)

**VIP**

(M=乙型肝炎肝硬化OR乙型病毒性肝炎肝硬化OR乙肝肝硬化)

AND

(M=中医OR中药OR中西医OR中医药OR方剂OR方药)

**SinoMed:**

("乙型肝炎肝硬化"[常用字段:智能] OR"乙型病毒性肝炎肝硬化"[常用字段:智能] OR"乙肝肝硬化"[常用字段:智能])

AND

("中医"[常用字段:智能] OR"中药"[常用字段:智能] OR"中西医"[常用字段:智能]OR"中医药"[常用字段:智能] OR"方剂"[常用字段:智能]OR "方药"[常用字段:智能])

**Pubmed**

(("Liver Cirrhosis"[Mesh]) OR ((Hepatic Cirrhosis) OR (Liver Cirrhosis) OR (Cirrhosis, Hepatic) OR (Cirrhosis, Liver) OR (Fibrosis, Liver) OR (Liver Fibrosis)))

AND

(("Medicine, Chinese Traditional"[Mesh]) OR ((Medicine, Chinese Traditional) OR (Zhong Yi Xue) OR (Chung I Hsueh) OR (Hsueh, Chung I) OR (Chinese Medicine, Traditional) OR (Chinese Traditional Medicine) OR (Traditional Chinese Medicine) OR (Traditional Medicine, Chinese) OR (Traditional Tongue Diagnosis) OR (Tongue Diagnoses, Traditional) OR (Tongue Diagnosis, Traditional) OR (Traditional Tongue Diagnoses) OR (Traditional Tongue Assessment) OR (Tongue Assessment, Traditional) OR (Traditional Tongue Assessments) OR (Herbal medicine) OR (herb-medicine) OR (Chinese herbal medicine) OR (Integrated traditional Chinese) OR (Complementary medicine) OR (Chinese medicine) OR (patent medicine) OR (herb)))

**Web of Science**

(TS=(Liver Cirrhosis) OR AB=(Hepatic Cirrhosis OR Liver Cirrhosis OR Cirrhosis, Hepatic OR Cirrhosis, Liver OR Fibrosis, Liver OR Liver Fibrosis))

AND

(TS=(Medicine, Chinese Traditional) OR AB=(Medicine, Chinese Traditional OR Zhong Yi Xue OR Chung I Hsueh OR Hsueh, Chung I OR Chinese Medicine, Traditional OR Chinese Traditional Medicine OR Traditional Chinese Medicine OR Traditional Medicine, Chinese OR Traditional Tongue Diagnosis OR Tongue Diagnoses, Traditional OR Tongue Diagnosis, Traditional OR Traditional Tongue Diagnoses OR Traditional Tongue Assessment OR Tongue Assessment, Traditional OR Traditional Tongue Assessments OR Herbal medicine OR herb-medicine OR Chinese herbal medicine OR Integrated traditional Chinese OR Complementary medicine OR Chinese medicine OR patent medicine OR herb))

**Cochrane library**

#1 (MeSH descriptor: [Liver Cirrhosis] explode all trees)

#2 (Liver Cirrhosis or Hepatic Cirrhosis or Cirrhosis, Hepatic or Cirrhosis, Liver or Fibrosis, Liver or Liver Fibrosis)

#3 #1 or #2

#4 (MeSH descriptor: [Medicine, Chinese Traditional] explode all trees)

#5 (Medicine, Chinese Traditional or Zhong Yi Xue or Chung I Hsueh or Hsueh, Chung I or Chinese Medicine, Traditional or Chinese Traditional Medicine or Traditional Chinese Medicine or Traditional Medicine, Chinese or Traditional Tongue Diagnosis or Tongue Diagnoses, Traditional or Tongue Diagnosis, Traditional or Traditional Tongue Diagnoses or Traditional Tongue Assessment or Tongue Assessment, Traditional or Traditional Tongue Assessments or Herbal medicine or herb-medicine or Chinese herbal medicine or Integrated traditional Chinese or Complementary medicine or Chinese medicine or patent medicine or herb)

#6 #4 or #5

#7 #3 and #6

**Embase**

#1 (hepatic cirrhosis OR liver cirrhosis OR cirrhosis, hepatic OR cirrhosis, liver OR fibrosis, liver OR liver fibrosis)

#2 (medicine, chinese traditional OR zhong yi xue OR chung i Hsueh OR hsueh, chung I OR chinese medicine, traditional OR chinese traditional medicine OR traditional chinese medicine OR traditional medicine, Chinese OR traditional tongue diagnosis OR tongue diagnoses, traditional OR tongue diagnosis, traditional OR traditional tongue diagnoses OR traditional tongue assessment OR tongue assessment, traditional OR traditional tongue assessments OR herbal medicine OR herb medicine OR chinese herbal medicine OR integrated traditional Chinese OR complementary medicine OR chinese medicine OR patent medicine OR herb)

#3 #1 and #2

**original literatures of decompensated hepatitis B cirrhosis**

| 益气逐瘀法治疗乙肝肝硬化腹水18例 | 张振海 | 1997 | 一贯煎治疗乙肝肝硬化腹水204例临床疗效观察 | 向丹沙 | 2015 |
| --- | --- | --- | --- | --- | --- |
| 消臌汤治疗乙肝后肝硬化腹水52例 | 张晓江 | 1999 | 中西医联合治疗失代偿期乙型肝炎肝硬化患者的疗效研究 | 胡建红 | 2016 |
| 中西医结合治疗肝硬化腹水 | 罗欣拉 | 2000 | 健脾化瘀利水方治疗乙肝后肝硬化腹水可行性研究 | 李晓杰 | 2016 |
| 上补下泻法治疗肝硬化腹水 50 例 | 杨炳洪 | 2001 | 血府逐瘀汤加减治疗10例乙型肝炎肝硬化脾亢患者脾介入栓塞术后发热的体会 | 程刚 | 2016 |
| 鳖甲三虫汤治疗乙肝后肝硬化腹水37例 | 胡晓峰 | 2001 | 保肝利水汤联合西药治疗乙肝肝硬化顽固性腹水临床观察 | 黄瑞华 | 2016 |
| 加味苓桂术甘汤治疗乙型肝炎后肝硬化腹水64例临床观察 | 陈兰玲 | 2001 | 中西医结合治疗乙肝后肝硬化腹水34例 | 程无为 | 2016 |
| 活血化瘀兼利水法治疗乙肝肝硬化初次腹水42例 | 卢裕兴 | 2001 | 中西医结合治疗乙肝后肝硬化腹水临床分析 | 高宏 | 2016 |
| 中西医结合治疗乙型肝炎后肝硬化腹水66例--附西药治疗52例对照 | 李超 | 2002 | 乙肝后肝硬化腹水的中西医结合治疗效果研究 | 韩方方 | 2016 |
| 中西医结合治疗肝硬化腹水46例 | 王敬碧 | 2002 | 中西医结合治疗乙肝后肝硬化腹水(气滞湿阻证)临床疗效分析 | 华鹏 | 2016 |
| 辨证施治肝硬化腹水50例 | 许院院 | 2003 | 浅析中医综合治疗乙肝肝硬化顽固性腹水临床效果 | 李士权 | 2016 |
| 加味四君五皮饮治疗乙型肝炎后肝硬化腹水30例总结 | 潘博 | 2004 | 中药口服联合西药常规治疗乙型肝炎肝硬化顽固性腹水20例 | 李晓华 | 2016 |
| 中西医结合治疗肝炎后肝硬化肝性胸水32例疗效观察 | 俞文军 | 2005 | 中医辨证治疗乙肝肝硬化腹水临床研究 | 尚杰云 | 2016 |
| 健肝汤治疗肝硬化失代偿期46例 | 姚传美 | 2005 | 中西医结合治疗乙肝肝硬化顽固性腹水的疗效 | 孙继红 | 2016 |
| 自拟鼓胀方治疗肝硬化腹水50例 | 张小兆 | 2005 | 加用自拟中药方治疗乙型肝炎肝硬化的临床观察 | 孙世长 | 2016 |
| 鳖甲汤治疗乙肝肝硬化腹水38例 | 杨杰 | 2005 | 肝宁方治疗乙型肝炎肝硬化合并腹水30例临床观察 | 覃黎葵 | 2016 |
| 贺普丁联合扶正活血解毒汤治疗乙型肝炎肝硬化失代偿期18例 | 葛忠良 | 2005 | 加味实脾饮治疗乙肝肝硬化顽固性腹水合并肝性胸水 | 徐祥涛 | 2016 |
| 软肝消水汤治疗肝硬化腹水35例临床观察 | 赵书超 | 2005 | 中西医结合治疗乙肝肝硬化顽固性腹水的疗效探讨 | 张乐刚 | 2016 |
| 30例肝硬化腹水中西医结合治疗体会 | 张树荣 | 2006 | 益气散结消臌汤治疗血瘀型乙型肝炎肝硬化腹水的疗效及对生活质量的影响 | 张金良 | 2017 |
| 中西医结合治疗乙型肝炎后肝硬化腹水59例总结 | 赵斌 | 2006 | 解毒化瘀柔肝利水法治疗肝硬化腹水31例 | 刘宏平 | 2017 |
| 中西医结合治疗乙型肝炎肝硬化腹水76例临床观察 | 石次国 | 2007 | 五苓散联合六味地黄丸加减治疗乙肝肝硬化腹水的疗效分析 | 林雨果 | 2017 |
| 扶正固本治疗肝炎后肝硬化腹水92例 | 范永军 | 2007 | 消臌汤结合西医疗法治疗乙肝肝硬化腹水患者的临床观察 | 王桂娟 | 2017 |
| 中西医结合治疗乙型肝炎肝硬化腹水76例临床观察 | 石次国 | 2007 | 中西医结合治疗肝硬化腹水临床效果观察 | 王磊 | 2017 |
| 复方甘草酸苷联合自拟中药汤剂治疗乙型肝炎肝硬化腹水38例临床观察 | 田发勋 | 2007 | 苍牛防己黄芪汤治疗乙型肝炎肝硬化腹水的临床分析 | 芦德银 | 2017 |
| 中西医结合治疗乙型肝炎后肝硬化腹水60例 | 李建明 | 2007 | 扶阳达肝汤治疗乙型病毒性肝炎后肝硬化腹水46例总结 | 陈思任 | 2017 |
| 鼓胀方治疗乙型肝炎肝硬化腹水50例临床观察 | 杨铁骊 | 2007 | 恩替卡韦联合自拟中药治疗失代偿期乙型肝炎肝硬化患者疗效初步研究 | 何伟 | 2017 |
| 软肝消癥利水汤治疗乙肝后肝硬化腹水34例 | 孙尚见 | 2007 | 中西医结合治疗肝硬化腹水的效果评价 | 贺东辉 | 2017 |
| 中西医结合治疗肝炎后肝硬化腹水并发出血倾向35例体会 | 畅辉军 | 2008 | 温阳健脾汤加减治疗乙肝肝硬化顽固性腹水临床研究 | 张健 | 2017 |
| 健脾泄浊化瘀汤治疗慢性乙型肝炎后肝硬化腹水(脾虚血瘀型)的临床观察 | 史海立 | 2008 | 五苓散加减联合西药治疗乙肝后肝硬化腹水30例疗效分析 | 王平 | 2017 |
| 疏肝健脾活血利水法治疗乙型肝炎后肝硬化腹水60例疗效观察 | 郎世平 | 2008 | 炙甘草汤合猪苓汤治疗乙型肝炎肝硬化腹水的临床疗效观察 | 刘礼剑 | 2017 |
| 温阳利水汤联合拉米夫定治疗脾肾阳虚型乙型肝炎肝硬化腹水疗效观察 | 王玉忠 | 2008 | 中西医结合治疗乙肝肝硬化顽固性腹水50例疗效观察 | 刘迎辉 | 2017 |
| 中西医结合治疗乙型肝炎肝硬化腹水疗效观察 | 杨怀书 | 2008 | 柔肝散结汤联合恩替卡韦治疗慢性乙肝肝硬化失代偿期的临床研究 | 柳志原 | 2017 |
| 益气化瘀消臌汤治疗肝硬化腹水58例 | 李日向 | 2008 | 乙型肝炎后肝硬化腹水运用中西医结合治疗的疗效观察 | 王花花 | 2017 |
| 中西医结合治疗肝硬化腹水31例观察 | 李克忠 | 2009 | 舒肝健脾汤对乙型肝炎肝硬化肠道菌群移位及腹水的影响 | 王志勇 | 2017 |
| 消臌强肝汤治疗血瘀型乙型肝炎肝硬化腹水的研究 | 梁宏 | 2009 | 乙肝后肝硬化腹水中医为主治疗临床探讨 | 杨成庆 | 2017 |
| 中药联合阿德福韦酯治疗乙型肝炎肝硬化失代偿期疗效观察 | 敬小华 | 2009 | 中药消臌汤联合西药治疗乙肝肝硬化腹水的临床观察 | 姚瑞 | 2017 |
| 中西医结合治疗肝炎后肝硬化腹水30例临床观察 | 骆洁恒 | 2009 | 清营汤加减对乙肝后肝硬化内毒素血症患者体内内毒素水平的影响 | 张春光 | 2017 |
| 中西医结合治疗失代偿期乙型肝炎肝硬化45例 | 马新社 | 2009 | 中西医结合治疗乙肝肝硬化顽固性腹水疗效分析 | 张华平 | 2017 |
| 中西医结合治疗乙肝后肝硬化腹水36例临床观察 | 欧阳祖新 | 2009 | 益气和血方联合恩替卡韦治疗乙肝肝硬化失代偿期脾功能亢进疗效及对免疫功能影响研究 | 钟军华 | 2017 |
| 复方草酸苷联合中药汤剂治疗乙肝肝硬化腹水 | 夏合中 | 2009 | 关于中西医结合治疗肝硬化腹水的效果评价 | 李彦丽 | 2017 |
| 真武汤治疗脾肾阳虚型乙肝肝硬化腹水30例 | 杨艳娜 | 2009 | 用猪苓汤合炙甘草汤治疗乙型肝炎所致肝硬化腹水的效果观察 | 丁杨斌 | 2018 |
| 中西医结合治疗慢性乙型肝炎合并肝硬化腹水24例 | 赵鹏 | 2009 | 中医辨证治疗肝硬变腹水56例 | 李广宣 | 2018 |
| 乙型肝炎肝硬化失代偿期抗病毒与中药联合治疗的临床观察 | 张军 | 2010 | 观察真武汤治疗脾肾阳虚型乙肝肝硬化腹水的疗效 | 杨菁 | 2018 |
| 消臌汤辅助治疗乙型肝炎肝硬化腹水的临床观察 | 周英栋 | 2010 | 乙肝后肝硬化腹水中医为主治疗临床分析 | 邓光远 | 2018 |
| 脾益肾活血利水法治疗失代偿期肝硬化32例临床观察 | 林景松 | 2010 | 对乙肝肝硬化顽固性腹水合并腹股沟疝患者进行中西医结合治疗的效果及安全性分析 | 傅涛 | 2018 |
| 清毒化瘀汤治疗湿热夹瘀型肝硬化失代偿期内毒素血症60例 | 郑琳 | 2011 | 实脾饮联合四君子汤治疗乙型肝炎肝硬化腹水临床观察 | 徐敬江 | 2018 |
| 益肝软坚升白汤联合恩替卡韦治疗慢性乙型肝炎肝硬化失代偿期临床观察 | 钱海清 | 2011 | 乙型肝炎后肝硬化腹水中医为主治疗的临床疗效观察 | 王强 | 2018 |
| 茯白方联合恩替卡韦分散片治疗乙型肝炎肝硬化低蛋白血症临床观察 | 史文丽 | 2011 | 益气和血方联合恩替卡韦片治疗乙型肝炎病毒相关肝硬化失代偿期气虚血瘀证患者35例临床观察 | 李攀 | 2018 |
| 中西医结合治疗失代偿期肝硬化90例临床观察 | 韩丽英 | 2011 | 自拟疏肝利水方治疗乙肝肝硬化腹水的临床观察 | 刘海艳 | 2018 |
| 中西医结合治疗乙肝肝硬化腹水临床观察 | 覃杰锋 | 2011 | 灸甘草汤合猪苓汤治疗乙型肝炎肝硬化腹水的临床疗效观察 | 刘树辉 | 2018 |
| 中西医结合治疗乙肝后肝硬化腹水54例疗效观察 | 尹艳雅 | 2012 | 茵陈术附汤合温胆汤加减治疗乙肝肝硬化腹水临床观察 | 骆彩英 | 2018 |
| 自拟鼓胀方治疗乙型肝炎肝硬化腹水效果观察 | 李白龙 | 2012 | 五苓散联合西药治疗乙型肝炎肝硬化腹水(气阴两虚)随机平行对照研究 | 宛瑞杰 | 2018 |
| 30例中西医结合治疗慢性乙型肝炎合并肝硬化腹水临床分析 | 汤英 | 2012 | 脾虚血瘀水停型乙肝肝硬化腹水患者采用健脾活血利水汤治疗的有效性及安全性 | 刘彦兴 | 2018 |
| 中西医结合治疗乙型肝炎后肝硬化腹水43例 | 王春安 | 2012 | 五苓散加减治疗乙肝肝硬化腹水临床疗效分析 | 徐佳娜 | 2018 |
| 中西医结合治疗肝硬化腹水80例 | 钟军华 | 2012 | 温阳健脾化瘀行水法配合白蛋白、血浆治疗乙肝后肝硬化腹水的临床观察 | 赵竟秀 | 2018 |
| 中西医结合治疗乙肝后肝硬化腹水56例临床观察 | 兰墨赭 | 2012 | 五苓散联合六味地黄丸对乙肝肝硬化腹水的治疗作用 | 周勋 | 2018 |
| 消臌汤治疗慢性乙型肝炎肝硬化腹水25例 | 彭卫 | 2012 | 疏肝理脾合剂治疗乙肝肝硬化腹水的临床疗效分析 | 张婕 | 2018 |
| 中西医结合治疗乙肝后肝硬化腹水临床观察 | 孙刚 | 2012 | 解郁软坚活血方联合西药治疗乙型肝炎肝硬化临床研究 | 张意兰 | 2018 |
| 中西医结合治疗乙肝肝硬化顽固性腹水 | 孙良秀 | 2012 | 益气活血方治疗乙型肝炎肝硬化腹水的临床研究 | 赵磊 | 2018 |
| 乙肝后肝硬化腹水中医为主治疗临床分析 | 王平军 | 2012 | 中西医结合对乙肝肝硬化顽固性腹水的临床分析 | 赵磊 | 2018 |
| 观察中西医治疗乙型肝炎后肝硬化腹水的临床效果 | 武果勤 | 2012 | 加减地黄饮子治疗乙型肝炎肝硬化肝性脑病临床效果观察 | 唐荣华 | 2019 |
| 中西药联合治疗肝硬化腹水50例疗效分析 | 刘崧 | 2013 | 西医疗法联合苍牛防己黄芪汤治疗乙肝肝硬化腹水的临床疗效 | 李一凡 | 2019 |
| 培元固本散治疗失代偿期肝硬化疗效观察 | 宋爱军 | 2013 | 扶正活血解毒汤联合恩替卡韦治疗乙肝肝硬化失代偿期临床观察 | 陈镇清 | 2019 |
| 疏肝化瘀利水汤治疗乙型肝炎后肝硬化腹水30例 | 李静 | 2013 | 真武汤治疗脾肾阳虚型乙肝肝硬化腹水的效果分析 | 王庆成 | 2019 |
| 中西医结合治疗乙型肝炎后肝硬化腹水45例 | 董尚丰 | 2013 | 自拟疏肝健脾化瘀汤联合大黄䗪虫丸治疗乙型肝炎后肝硬化腹水临床观察 | 高玉杰 | 2019 |
| 中西医结合治疗乙肝肝硬化顽固性腹水临床探析 | 蒋敏 | 2013 | 中西医结合治疗乙肝肝硬化腹水50例临床观察 | 李洁 | 2019 |
| 中西医结合治疗乙肝肝硬化顽固性腹水临床分析 | 陈剑 | 2013 | 中西医结合治疗对乙型肝炎肝硬化失代偿期患者外周血Th/Treg平衡及淋巴细胞的影响研究 | 黄风雷 | 2019 |
| 乙肝肝硬化失代偿期中药治疗的临床探讨 | 黄耀龙 | 2013 | 茵陈术附汤合温胆汤加减方治疗乙型病毒性肝炎所致肝硬化腹水的疗效分析 | 曹琪 | 2019 |
| 中西医结合治疗乙型肝炎后肝硬化腹水疗效观察 | 邓雪梅 | 2013 | 通络解毒方联合恩替卡韦治疗乙肝肝硬化失代偿期临床研究 | 陈军 | 2019 |
| 中西医结合治疗乙型肝炎后肝硬化腹水 | 李进东 | 2013 | 炙甘草汤合猪苓汤治疗乙型肝炎肝硬化腹水临床观察 | 潘光勇 | 2019 |
| 双味泽苓合剂治疗乙型肝炎后肝硬化腹水气滞湿阻型33例 | 李严生 | 2013 | 益气活血方治疗乙型肝炎肝硬化腹水的疗效及对肝功能的影响 | 刘成彬 | 2019 |
| 乙肝后肝硬化腹水中医为主治疗临床分析 | 刘日媛 | 2013 | 中西医结合治疗乙型肝炎后肝硬化腹水33例临床观察 | 赵义红 | 2019 |
| 中西医结合治疗乙型肝炎后肝硬化腹水35例 | 刘悦明 | 2013 | 中西医结合治疗乙肝肝硬化腹水30例临床观察 | 马拯华 | 2019 |
| 中西医结合治疗肝硬化腹水54例疗效观察 | 罗艺徽 | 2013 | 恩替卡韦联合自拟中药治疗失代偿期乙型病毒性肝炎肝硬化患者的疗效观察 | 赵生珍 | 2019 |
| 中西药联合治疗失代偿期乙型肝炎肝硬化30例 | 苏立稳 | 2013 | 中西医结合治疗乙肝肝硬化顽固性腹水的临床体会 | 钟晓慧 | 2019 |
| 恩替卡韦联合复方肝欣合剂治疗失代偿期乙型肝炎肝硬化临床研究 | 苏立稳 | 2013 | 西医疗法联合苍牛防己黄芪汤治疗乙肝肝硬化腹水的临床疗效 | 王月莹 | 2019 |
| 中西医结合治疗乙型肝炎后肝硬变腹水30例 | 李静 | 2013 | 益气舒肝方辅治乙肝肝硬化腹水临床观察 | 魏晓梅 | 2020 |
| 五苓散加减联合西药治疗乙肝后肝硬化腹水56例 | 赵金豹 | 2013 | 一贯煎加味联合西药治疗乙型病毒性肝炎肝硬化腹水的临床观察 | 常征斌 | 2020 |
| 鼓胀消治疗乙型肝炎后肝硬化腹水44例 | 钟婉婷 | 2013 | 评价注射用谷胱甘肽联合中药汤剂治疗乙肝肝硬化腹水的效果 | 李丽 | 2020 |
| 滋阴养血法对肝硬化失代偿的疗效观察及肝脏合成功能的影响 | 张来 | 2013 | 中西医结合治疗慢性乙型肝炎后肝硬化腹水疗效分析 | 陈姝霞 | 2020 |
| 中西医结合治疗肝硬化腹水52例疗效观察 | 刘剑辉 | 2014 | 解毒化瘀健脾方治疗慢性乙型肝炎肝硬化腹水的临床观察 | 陈霞 | 2020 |
| 中西医结合治疗乙型肝炎后肝硬化腹水疗效探讨 | 韩淑玲 | 2014 | 中西医结合治疗肝硬化腹水的临床疗效观察 | 黄勇 | 2020 |
| 中西药结合治疗失代偿期乙肝肝硬化的疗效 | 李正良 | 2014 | 四合饮治疗乙型病毒性肝炎肝硬化腹水临床研究 | 宋爱军 | 2020 |
| 中西医结合治疗乙肝肝硬化顽固性腹水 | 李浩 | 2014 | 五苓散加减治疗乙肝肝硬化腹水临床观察 | 陈敏 | 2020 |
| 中西医结合治疗乙型肝炎后肝硬化腹水临床疗效分析 | 杨文忠 | 2014 | 芪胡消癥汤佐治肝郁脾虚型乙肝肝硬化门静脉高压腹水42例观察 | 毛焕荣 | 2020 |
| 猪苓汤联合西药治疗乙型肝炎后肝硬化腹水的临床疗效观察 | 沈杰 | 2014 | 益气化浊解胀汤治疗乙肝肝硬化失代偿期的临床疗效及对谷草转氨酶-血小板比值指数的影响 | 蔡翠珠 | 2020 |
| 中西医结合治疗乙型肝炎后肝硬化腹水研究 | 朱琼香 | 2014 | 炙甘草汤合猪苓汤治疗乙型肝炎肝硬化腹水的临床疗效观察 | 翟雪珍 | 2020 |
| 中西医结合治疗乙肝肝硬化顽固性腹水临床探析 | 惠广进 | 2014 | 中药联合替诺福韦酯治疗乙肝肝硬化腹水疗效观察 | 董桂芬 | 2020 |
| 消臌汤治疗乙肝肝硬化腹水疗效观察 | 李家祥 | 2014 | 健脾活血利水汤治疗乙肝肝硬化腹水临床观察 | 郭师 | 2020 |
| 中西医结合治疗乙肝肝硬化腹水22例临床观察 | 李勇华 | 2014 | 和血调肝汤联合恩替卡韦治疗气滞血瘀型失代偿期乙肝肝硬化 | 郭杏斐 | 2020 |
| 扶阳利水方治疗慢性乙型肝炎后肝硬化腹水临床研究 | 凌家生 | 2014 | 中西医结合治疗慢性乙型病毒性肝炎后肝硬化腹水的疗效 | 刘旭辉 | 2020 |
| 健脾化瘀利水方治疗乙肝后肝硬化腹水110例临床观察 | 钱静燕 | 2014 | 为乙型肝炎后肝硬化腹水患者使用健脾化臌汤联合西医疗法进行治疗的效果 | 牛艳 | 2020 |
| 中西医结合治疗乙肝肝硬化顽固性腹水患者的临床疗效评估 | 任志强 | 2014 | 壮方柔肝化纤颗粒治疗肝肾阴虚型乙肝肝硬化腹水的效果 | 吴姗姗 | 2020 |
| 78例替比夫定中西医治疗失代偿期乙肝肝硬化的临床疗效分析 | 王全权 | 2014 | 运用益气解毒通络法治疗乙肝肝硬化失代偿期 | 许海芹 | 2020 |
| 中西药联合治疗肝病的临床观察 | 王世利 | 2014 | 中西医结合治疗乙型肝炎肝硬化顽固性腹水 | 杨建辉 | 2020 |
| 中西医结合治疗乙肝肝硬化顽固性腹水的临床体会 | 叶美琼 | 2014 | 健脾疏肝汤加减联合恩替卡韦治疗乙肝肝硬化失代偿期的效果 | 张宇 | 2020 |
| 中西医治疗慢性乙型肝炎肝硬化的效果观察 | 赵娟 | 2014 | 中西医结合治疗乙肝肝硬化失代偿期脾功能亢进33例临床观察 | 邹俊锋 | 2020 |
| 中西医结合治疗乙型肝炎后肝硬变腹水300例 | 朱运华 | 2014 | 健脾利水汤联合西医常规治疗乙型病毒性肝炎肝硬化30例 | 李晓华 | 2021 |
| 中西医结合治疗乙型肝炎后肝硬化腹水疗效观察 | 孙晓峰 | 2014 | 益气养血、通络解毒法联合抗病毒治疗对乙型肝炎肝硬化失代偿期患者肝功能及生活质量的影响 | 李慧 | 2021 |
| 中西医结合治疗乙肝肝硬化顽固性腹水60例临床观察 | 卓科 | 2014 | 健脾利水方治疗中重度乙型肝炎肝硬化腹水的临床观察 | 赵小丽 | 2021 |
| 软肝汤联合恩替卡韦治疗乙肝肝硬化失代偿期的临床研究 | 于征 | 2015 | 柔肝化纤颗粒治疗乙肝肝硬化腹水的临床疗效及对肝纤维化、氧化应激水平的影响 | 吕艳杭 | 2021 |
| 拉米夫定结合补阳还五汤治疗乙型肝炎肝硬化代偿期55例临床观察 | 勾文忠 | 2015 | 中西医结合治疗乙型肝炎肝硬化腹水30例临床观察 | 赵小红 | 2021 |
| 健脾化臌汤联合西药治疗乙型肝炎后肝硬化腹水临床疗效 | 李晓利 | 2015 | 健脾活血利水方对脾虚水停证乙型肝炎肝硬化腹水疗效及T淋巴细胞亚群影响 | 张莉莉 | 2021 |
| 中西医结合治疗乙型肝炎后肝硬化腹水的临床疗效 | 汤刚义 | 2015 | 温阳健脾方联合西医常规疗法治疗乙型肝炎肝硬化失代偿期患者的研究 | 陈颖琪 | 2021 |
| 中西医结合治疗乙肝肝硬化顽固性腹水效果观察 | 孙尚洪 | 2015 | 健脾利水汤联合西医常规治疗乙型病毒性肝炎肝硬化30例 | 李晓华 | 2021 |
| 中西医结合治疗乙型肝炎肝硬变腹水72例 | 王玉忠 | 2015 | 中西医结合治疗脾虚水停型乙肝肝硬化腹水31例 | 林震群 | 2021 |
| 清热除湿、理气散满法治疗肝硬化腹水的临床研究 | 胡青海 | 2015 | 炙甘草汤合猪苓汤对乙型肝炎肝硬化腹水患者肝功能及凝血功能的影响 | 刘安勇 | 2021 |
| 益气活血利水法治疗乙肝肝硬化初次腹水疗效观察 | 邓静 | 2015 | 血府逐瘀汤加减对乙肝肝硬化失代偿期肝纤维化及炎性因子的影响 | 刘巍 | 2021 |
| 自拟鼓胀方治疗乙型肝炎肝硬化腹水 | 陈忠瑞 | 2015 | 柴胡当归散治疗少阳太阴合病证乙肝肝硬化腹水 | 阮博文 | 2021 |
| 中西医结合治疗乙肝肝硬化顽固性腹水临床研究 | 朱雪 | 2015 | 五苓散联合六味地黄丸治疗乙肝肝硬化腹水临床观察 | 李兆阳 | 2021 |
| 中西医结合治疗乙肝肝硬化顽固性腹水的临床效果观察 | 郭志勇 | 2015 | 柴胡桂枝干姜汤加味治疗慢性乙型肝炎失代偿期肝硬化残留黄疸临床观察 | 韦璐莹 | 2021 |
| 中药汤剂联合西医治疗肝硬化顽固性腹水临床研究 | 陈继林 | 2015 | 益气和血方治疗乙肝肝硬化失代偿期脾功能亢进患者的临床效果及对脾脏功能的影响 | 张胥磊 | 2021 |
| 健脾化痰凉血活血利水方治疗乙型肝炎肝硬化腹水临床疗效探讨 | 蒋岩 | 2015 | 育阴利水膏治疗乙肝肝硬化腹水临床观察 | 朱沪敏 | 2021 |
| 益气健脾活血利水汤联合西药治疗乙肝后肝硬化腹水随机平行对照研究 | 林树煌 | 2015 | 胃苓软肝消胀散治疗乙肝后肝硬化腹水的临床研究 | 关红孝 | 2022 |
| 中西医结合治疗乙肝肝硬化顽固性腹水临床效果分析 | 罗华兵 | 2015 | 健脾化湿止血方对乙肝肝硬化食管胃静脉曲张破裂出血患者二级预防的随机对照研究 | 侯艺鑫 | 2022 |
| 中西医结合治疗乙型肝炎肝硬化腹水的临床观察 | 谢函君 | 2015 | 膈下逐瘀汤合六君子汤治疗乙肝肝硬化腹水临床观察 | 李璐璐 | 2022 |
| 中西医结合治疗慢性乙型肝炎合并肝硬化腹水的临床疗效分析 | 张华平 | 2015 | 猪苓汤加减联合西药治疗乙型病毒性肝炎肝硬化腹水临床研究 | 林军 | 2022 |
| 中西医结合治疗乙肝肝硬化顽固性腹水的临床观察 | 周怀兵 | 2015 | 苍牛防己黄芪汤辅治乙肝肝硬化腹水临床观察 | 王海涛 | 2022 |
|  |  |  | 膈下逐瘀汤加减联合西药治疗乙肝肝硬化临床研究 | 严秀芝 | 2022 |

**original literatures of compensated hepatitis B cirrhosis**

| 拉米夫定合中药治疗慢性乙型肝炎并早期肝硬化29例 | 范恒 | 2003 | 化瘀软肝汤对代偿期乙型肝炎肝硬变患者肝脏功能及凝血功能的影响 | 余晓珂 | 2018 |
| --- | --- | --- | --- | --- | --- |
| 辨证应用疏肝化瘀颗粒治疗代偿期乙肝肝硬化临床研究 | 邵国辉 | 2010 | 复肝汤联合恩替卡韦治疗乙型病毒性肝炎肝硬化代偿期临床研究 | 张锐 | 2018 |
| 中西医结合治疗早期肝硬化的临床疗效 | 陈卫兵 | 2011 | 丹鸡活血汤联合恩替卡韦片治疗慢性乙型肝炎肝硬化的疗效及对患者血清炎症因子和肝功能的影响 | 周晶 | 2018 |
| 一贯煎联合阿德福韦酯治疗慢性乙型肝炎早期肝硬化25例 | 李爱芝 | 2012 | 逐瘀正肝汤对乙肝肝硬化患者血流动力学及肝纤维化的影响 | 车军勇 | 2018 |
| 中西医结合治疗乙型肝炎早期肝硬化临床疗效研究 | 汤英 | 2012 | 益气祛瘀饮联合多烯磷脂酰胆碱治疗慢性乙肝早期肝硬化疗效及对肝纤维化指标、炎性细胞因子的影响 | 谈晓洁 | 2019 |
| 中西医结合治疗148例乙型肝炎早期肝硬化 | 柴燕萍 | 2012 | 通络消癥汤治疗乙肝肝硬化代偿期(肝郁脾虚夹瘀证)对肝功能及PCⅢ、LN水平的影响 | 陈军 | 2019 |
| 柴芍六君水蛭汤治疗乙型肝炎后肝硬化代偿期48例疗效观察 | 陈利平 | 2012 | 丹参川芎嗪合养血柔肝组方治疗慢性乙型肝炎早期肝硬化 | 何倩丹 | 2019 |
| 补肾化瘀方联合恩替卡韦治疗乙型肝炎肝硬化代偿期疗效观察 | 丁锷 | 2012 | 乙型肝炎肝硬化代偿期采用软肝化瘀汤的临床效果分析 | 黄迎春 | 2019 |
| 益气破血行气通络中药对乙型肝炎代偿期肝硬化患者的治疗作用 | 王仲霞 | 2012 | 恩替卡韦联合化瘀软肝汤治疗乙型肝炎肝硬化代偿期患者51例 | 刘雷 | 2019 |
| 理脾法治疗乙肝肝硬化代偿期的临床观察 | 张凤敏 | 2012 | 中医化瘀软肝汤对代偿期乙型肝炎肝硬变患者肝脏功能、凝血功能的影响研究 | 陆娇 | 2019 |
| 阿德福韦酯联合柴胡疏肝散治疗乙型肝炎后早期肝硬化70例 | 郭占芳 | 2013 | 一贯煎加味辅助治疗HBeAg阴性慢性乙型肝炎活动性代偿期肝硬化的效果分析 | 邱腾宇 | 2019 |
| 下瘀血汤加味治疗早期肝硬化45例 | 陈新华 | 2013 | 中医药干预对代偿期乙型肝炎肝硬化患者影响分析 | 任凤娜 | 2019 |
| 新拟和肝丸加减治疗慢性乙型肝炎代偿期肝硬化32例 | 徒康宛 | 2013 | 抑木扶土法联合恩替卡韦治疗代偿期乙肝肝硬化疗效及对肝功能和肝纤维化指标的影响 | 邵小梅 | 2019 |
| 替比夫定联合活血通络方治疗乙型肝炎肝硬化代偿期临床观察 | 冯淑涵 | 2013 | 健脾疏肝法治疗乙肝肝硬化代偿期肝郁脾虚证临床观察 | 覃婕 | 2019 |
| 中西医结合治疗慢性乙肝早期肝硬化34例临床分析 | 梁文萍 | 2013 | 通络消癥汤治疗乙肝肝硬化代偿期肝郁脾虚夹瘀证临床分析 | 陶学山 | 2019 |
| 中西医结合治疗慢性乙型肝炎早期肝硬化临床疗效观察 | 王贞 | 2013 | 疏肝健脾汤联合恩替卡韦治疗肝郁脾虚型代偿期乙肝肝硬化临床观察 | 王宏飞 | 2019 |
| 化瘀软肝汤治疗代偿期乙型肝炎肝硬化的疗效观察 | 张云华 | 2014 | 通络消癥汤治疗乙肝肝硬化代偿期肝郁脾虚夹瘀证临床疗效 | 王敬 | 2019 |
| 甘露消毒丹联合恩替卡韦治疗代偿期乙肝肝硬化25例 | 彭琳 | 2014 | 柴胡软肝散治疗乙肝早期肝硬化(肝脾不调型)的临床研究 | 王伟东 | 2019 |
| 柴胡疏肝散联合抗病毒药物治疗CBD相关性代偿期肝硬化的观察 | 黄正桥 | 2015 | 代偿期乙型肝炎肝硬化恩替卡韦联合中药治疗临床观察 | 于延锋 | 2019 |
| 中西医结合治疗乙型肝炎并早期肝硬化的临床效果 | 黄光艳 | 2015 | 中西医结合对代偿期乙肝肝硬化气虚血瘀型的治疗效果观察 | 于媛 | 2019 |
| 益气软肝汤联合西药治疗乙肝后肝硬化代偿期随机平行对照研究 | 文驰 | 2015 | 通络消癥汤治疗乙肝肝硬化代偿期肝郁脾虚夹瘀证疗效观察 | 周文锋 | 2019 |
| 通络消癥汤治疗乙肝肝硬化代偿期肝郁脾虚夹瘀证的临床观察 | 陈兰玲 | 2015 | 中药软肝方改善乙型肝炎肝硬化肝纤维化的临床疗效观察 | 余思邈 | 2019 |
| 养阴化瘀汤联合西药治疗慢性乙型肝炎早期肝硬化随机平行对照研究 | 彭华明 | 2015 | 研究中西医结合的方式对代偿期乙肝肝硬化气虚血瘀型的临床治疗效果 | 梅前垒 | 2020 |
| 益气破血行气通络方联合恩替卡韦治疗乙型肝炎代偿期肝硬化60例 | 王灵广 | 2015 | 柴苓汤联合恩替卡韦治疗肝郁脾虚型乙肝肝硬化患者的临床疗效及对门脉血流动力学和血管内皮生成因子的影响 | 陈鹏兰 | 2020 |
| 解郁软坚活血颗粒联合西药治疗乙型肝炎代偿期肝硬化86例临床观察 | 张朝阳 | 2015 | 逍遥散联合恩替卡韦治疗肝郁脾虚型乙肝后肝硬化代偿期30例临床研究 | 葛冰景 | 2020 |
| 理脾法治疗乙肝肝硬化代偿期的临床效果分析 | 祝兆民 | 2015 | 益气活血法联合抗病毒药物对早期乙型肝炎肝硬化患者免疫功能和肝纤维化指标水平的影响 | 郝亚娟 | 2020 |
| 逍遥散加减治疗代偿期乙型肝炎肝硬变30例 | 郭百涛 | 2016 | 培土化癥汤辅助治疗乙型肝炎肝硬化代偿期临床研究 | 李琤 | 2020 |
| 中西医结合治疗早期乙肝肝硬化的疗效观察 | 岑成灿 | 2016 | 调肝化纤丸联合恩替卡韦治疗乙肝肝硬化代偿期疗效分析 | 李念 | 2020 |
| 一贯煎加味联合阿德福韦酯片治疗HBeAg阴性慢性乙型肝炎活动性代偿期肝硬化患者的疗效观察 | 段淑红 | 2016 | 评价中医通络消癥汤治疗乙肝肝硬化代偿期肝郁脾虚夹瘀证的临床疗效 | 李小红 | 2020 |
| 温脾疏肝汤联合西药治疗慢性乙型肝炎后肝硬化随机平行对照研究 | 肖德梅 | 2016 | 软肝汤联合阿德福韦酯片治疗代偿期乙型病毒性肝炎肝硬变30例 | 李媛 | 2020 |
| 甘草酸二铵联合中药治疗乙肝早期肝硬化80例临床观察 | 付同娟 | 2017 | 用中西医结合疗法治疗慢性乙型病毒性肝炎早期肝硬化的效果观察 | 牟帅 | 2020 |
| 益气破血行气通络中药对乙型肝炎代偿期肝硬化患者的治疗作用探析 | 董继刚 | 2017 | 疏肝健脾汤联合恩替卡韦治疗肝郁脾虚型代偿期乙型肝炎肝硬化的临床疗效 | 王希 | 2020 |
| 通络消癥汤治疗乙肝肝硬化代偿期肝郁脾虚夹瘀证的临床观察 | 郝慧敏 | 2017 | 化瘀软肝汤对代偿期乙型病毒性肝炎肝硬化的疗效分析 | 殷光辉 | 2020 |
| 中药滋阴活血汤配合阿德福韦酯治疗代偿期乙型肝炎肝硬化136例分析 | 章新成 | 2017 | 评价通络消癥汤治疗乙肝肝硬化(代偿期)肝郁脾虚夹瘀证的效果 | 邝敏 | 2021 |
| 恩替卡韦联合自拟方剂治疗乙肝肝硬化的效果 | 段成颖 | 2017 | 疏肝健脾汤联合恩替卡韦对肝郁脾虚型代偿期乙肝肝硬化患者肝功能的治疗效果 | 郭文强 | 2021 |
| 恩替卡韦联合自拟中药治疗代偿期乙型肝炎肝硬化患者对血清细胞因子水平的影响 | 李红哲 | 2017 | 疏肝健脾解毒化湿方合大黄蟅虫丸治疗乙肝代偿期肝硬化临床观察 | 张金付 | 2021 |
| 柴胡桂枝汤化裁治疗代偿期乙肝肝硬化临床观察 | 颜幸杰 | 2017 | 软肝化瘀饮辅助恩替卡韦分散片治疗慢性乙型肝炎肝硬化代偿期瘀血阻络证临床研究 | 边倩 | 2021 |
| 膈下逐瘀汤联合西药治疗乙型肝炎肝硬化代偿期临床观察 | 张金良 | 2017 | 恩替卡韦联合中药治疗乙型肝炎早期肝硬化的疗效分析 | 陈丹丹 | 2021 |
| 养血柔肝方治疗乙型肝炎肝硬化早期的临床研究 | 张伟 | 2018 | 分析通络消癥汤联合恩替卡韦分散片对代偿期乙型病毒性肝炎肝硬化患者的疗效 | 崔胜忠 | 2021 |
| 慢性乙肝早期肝硬化给予中西医结合治疗的效果评价 | 肖珍 | 2018 | 柔肝化纤颗粒对代偿期乙肝肝硬化肝肾阴虚型患者外周血NLRP3炎症小体及其产物表达的影响 | 段桂姣 | 2021 |
| 通络消癥汤对乙肝肝硬化(代偿期)肝郁脾虚夹瘀证治疗效果分析 | 陈继春 | 2018 | 软肝化纤汤治疗对乙型肝炎肝硬化代偿期患者肝功能的影响 | 李志恒 | 2021 |
| 代偿期乙肝肝硬化给予恩替卡韦及柴胡桂枝汤化裁的临床效果研究 | 丛鸿浩 | 2018 | 和血调肝汤治疗代偿期乙肝后肝硬化临床观察 | 梁国强 | 2021 |
| 中西医结合治疗乙肝后肝硬化对血清肝纤维化指标及肝功能的影响 | 高倩 | 2018 | 调肝化纤丸在乙肝肝硬化治疗中的应用及远期效果随访研究 | 彭云鹤 | 2021 |
| 中西医结合治疗乙型肝炎肝硬化代偿期临床观察 | 陈慧基 | 2018 | 疏肝健脾活血方对乙肝肝硬化临床疗效的探究 | 张千 | 2021 |
| 益气解毒通络方联合拉米夫定治疗代偿期乙型肝炎肝硬化疗效观察 | 金玉林 | 2018 | 疏肝健脾方联合恩替卡韦治疗肝郁脾虚证乙肝肝硬化患者的临床效果 | 刘峰 | 2021 |
| 中医药干预对代偿期乙型肝炎肝硬化患者生活质量的影响 | 刘瑜 | 2018 | 通络消癥汤治疗乙肝肝硬化代偿期肝郁脾虚夹瘀证患者的效果 | 胡志刚 | 2022 |
| 中西医联合治疗代偿期乙型肝炎肝硬化的临床研究 | 马玉美 | 2018 | 顾氏健脾益气柔肝方联合恩替卡韦对代偿期乙肝肝硬化患者疗效及肠道菌群和肠黏膜屏障功能的影响 | 沈灵娜 | 2022 |
| 通络消癥汤对乙肝肝硬化代偿期肝郁脾虚夹瘀证的治疗效果 | 杨菁 | 2018 |  |  |  |

**Supplementary File S2. High frequency herbs for compensated hepatitis B cirrhosis**

| **Chinese name** | **Latin name** | **Flavor** | **Property** | **Meridian** | **Frequency** | **Proportion** |
| --- | --- | --- | --- | --- | --- | --- |
| Huzhang | *POLYGONI CUSPIDATI RHIZOMA ET RADIX* | Bitter | Cold | Liver, Gallbladder, Lung | 10 | 0.93% |
| Maidong | *OPHIOPOGONIS RADIX* | Sweet, Bitter | Cold | Heart, Lung, Stomach | 10 | 0.93% |
| Baihuasheshecao | *HERBA HEDYOTIS* | Sweet, Weak | Cool | Stomach, Large intestine, Small intestine | 11 | 1.02% |
| Banxia | *PINELLIAE RHIZOMA* | Pungent | Warm | Spleen, Stomach, Lung | 11 | 1.02% |
| Niuxi | *ACHYRANTHIS BIDENTATAE RADIX* | Bitter, Sweet, Sour | Mild | Liver, Kidney | 11 | 1.02% |
| Rendongteng | *LONICERAE JAPONICAE CAULIS* | Sweet | Cold | Lung, Stomach | 11 | 1.02% |
| Yinchen | *ARTEMISIAE SCOPARIAE HERBA* | Bitter, Pungent | Cold | Spleen, Stomach, Liver, Gallbladder | 11 | 1.02% |
| Dilong | *PHERETIMA* | Salty | Cold | Liver, Spleen, Bladder | 12 | 1.12% |
| Jineijin | *GALL1 GIGERII ENDOTHELIUM CORNEUM* | Sweet | Mild | Spleen, Stomach, Small intestine, Bladder | 12 | 1.12% |
| Chenpi | *CITRI RETICULATAE PERICARPIUM* | Bitter, Pungent | Warm | Lung, Spleen | 13 | 1.21% |
| Chuanxiong | *CHUANXIONG RHIZOMA* | Pungent | Warm | Liver, Gallbladder, Pericardium | 13 | 1.21% |
| Gouqizi | *LYCII FRUCTUS* | Sweet | Mild | Liver, Kidney | 13 | 1.21% |
| San leng | *SPARGANII RHIZOMA* | Pungent, Bitter | Mild | Liver, Spleen | 13 | 1.21% |
| Taizishen | *PSEUDOSTELLARIAE RADIX* | Sweet, Bitter | Mild | Spleen, Lung | 14 | 1.30% |
| Zelan | *LYCOPI HERBA* | Bitter, Pungent | Warm | Liver, Spleen | 14 | 1.30% |
| Chuanshanjia | *MANIS SQUAMA* | Salty | Cold | Liver, Stomach | 15 | 1.40% |
| Dangshen | *CODONOPSIS RADIX* | Sweet | Mild | Spleen, Lung | 16 | 1.49% |
| Jixueteng | *SPATHOLOBI CAULIS* | Bitter, Sweet | Warm | Liver, Kidney | 16 | 1.49% |
| Dihuang | *REHMANNIAE RADIX* | Sweet, Bitter | Cold | Heart, Liver, Kidney | 16 | 1.49% |
| Tu bie chong | *EUPOLYPHAGA STELEOPHAGA* | Salty | Cold | Liver | 16 | 1.49% |
| Zhiqiao | *AURANTII FRUCTUS* | Bitter, Pungent, Sour | Cold | Spleen, Stomach | 16 | 1.49% |
| Ezhu | *CURCUMAE RHIZOMA* | Pungent, Bitter | Warm | Liver, Spleen | 18 | 1.67% |
| Yujin | *CURCUMAE RADIX* | Pungent, Bitter | Cold | Liver, Heart, Lung | 22 | 2.05% |
| Chishao | *PAEONIAERADIX RUBRA* | Bitter | Cold | Liver | 23 | 2.14% |
| Taoren | *PERSICAE SEMEN* | Bitter, Sweet | Mild | Heart, Liver, Large intestine | 25 | 2.33% |
| Gancao | *GLYCYRRHIZAE RADIX ET RHIZOMA* | Sweet | Mild | Heart, Lung, Spleen, Stomach | 37 | 3.44% |
| Danggui | *ANGELICAE SINENSIS RADIX* | Sweet, Pungent | Warm | Liver, Heart, Spleen | 40 | 3.72% |
| Huangqi | *ASTRAGALI RADIX* | Sweet | Warm | Lung, Spleen | 43 | 4.00% |
| Fuling | *PORIA* | Sweet, Weak | Mild | Heart, Lung, Spleen, Kidney | 49 | 4.56% |
| Baizhu | *ATRACTYLODIS MACROCEPHALAE RHIZOMA* | Bitter, Sweet | Warm | Spleen, Stomach | 53 | 4.93% |
| Biejia | *TRIONYCIS CARAPAX* | Salty | Cold | Liver, Kidney | 53 | 4.93% |
| Chaihu | *BUPLEURI RADIX* | Pungent, Bitter | Cold | Liver, Gallbladder, Lung | 54 | 5.02% |
| Danshen | *SALVIAE MILTIORRHIZAE RADIX ET RHIZOMA* | Bitter | Cold | Heart, Liver | 54 | 5.02% |
| Baishao | *PAEONIAE RADIX ALBA* | Bitter, Sour | Cold | Liver, Spleen | 58 | 5.40% |

**Supplementary File S3. High frequency herbs for decompensated hepatitis B cirrhosis**

| **Chinese name** | **Latin name** | **Flavor** | **Property** | **Meridian** | **Frequency** | **Proportion** |
| --- | --- | --- | --- | --- | --- | --- |
| Fuling | *PORIA* | Sweet, Weak | Mild | Heart, Lung, Spleen, Kidney | 173 | 5.85% |
| Huangqi | *ASTRAGALI RADIX* | Sweet | Warm | Lung, Spleen | 166 | 5.61% |
| Baizhu | *ATRACTYLODIS MACROCEPHALAE RHIZOMA* | Bitter, Sweet | Warm | Spleen, Stomach | 163 | 5.51% |
| Danshen | *SALVIAE MILTIORRHIZAE RADIX ET RHIZOMA* | Bitter | Cold | Heart, Liver | 122 | 4.12% |
| Biejia | *TRIONYCIS CARAPAX* | Salty | Cold | Liver, Kidney | 107 | 3.62% |
| Zexie | *ALISMATIS RHIZOMA* | Sweet, Weak | Cold | Kidney, Bladder | 107 | 3.62% |
| Gancao | *GLYCYRRHIZAE RADIX ET RHIZOMA* | Sweet | Mild | Heart, Lung, Spleen, Stomach | 95 | 3.21% |
| Danggui | *ANGELICAE SINENSIS RADIX* | Sweet, Pungent | Warm | Liver, Heart, Spleen | 84 | 2.84% |
| Chaihu | *BUPLEURI RADIX* | Pungent, Bitter | Cold | Liver, Gallbladder, Lung | 78 | 2.64% |
| Zhuling | *POLYPORUS* | Sweet, Weak | Mild | Kidney, Bladder | 78 | 2.64% |
| Da fu pi | *ARECAE PERICARPIUM* | Pungent | Warm | Spleen, Stomach, Large intestine, Small intestine | 73 | 2.47% |
| Chishao | *PAEONIAERADIX RUBRA* | Bitter | Cold | Liver | 68 | 2.30% |
| Dangshen | *CODONOPSIS RADIX* | Sweet | Mild | Spleen, Lung | 60 | 2.03% |
| Zelan | *LYCOPI HERBA* | Bitter, Pungent | Warm | Liver, Spleen | 53 | 1.79% |
| Taoren | *PERSICAE SEMEN* | Bitter, Sweet | Mild | Heart, Liver, Large intestine | 52 | 1.76% |
| Baishao | *PAEONIAE RADIX ALBA* | Bitter, Sour | Cold | Liver, Spleen | 51 | 1.72% |
| Cheqianzi | *PLANTAGINIS SEMEN* | Sweet | Cold | Liver, Kidney, Lung, Small intestine | 44 | 1.49% |
| Yinchen | *ARTEMISIAE SCOPARIAE HERBA* | Bitter, Pungent | Cold | Spleen, Stomach, Liver, Gallbladder | 45 | 1.52% |
| Yujin | *CURCUMAE RADIX* | Pungent, Bitter | Cold | Liver, Heart, Lung | 44 | 1.49% |
| Chenpi | *CITRI RETICULATAE PERICARPIUM* | Bitter, Pungent | Warm | Lung, Spleen | 40 | 1.35% |
| Guizhi | *CINNAMOMI RAMULUS* | Pungent, Sweet | Warm | Heart, Lung, Bladder | 37 | 1.25% |
| Chuanxiong | *CHUANXIONG RHIZOMA* | Pungent | Warm | Liver, Gallbladder, Pericardium | 33 | 1.12% |
| Houpo | *MAGNOLIAE OFFICINALIS CORTEX* | Bitter, Pungent | Warm | Spleen, Stomach, Lung, Large intestine | 31 | 1.05% |
| Jineijin | *GALL1 GIGERII ENDOTHELIUM CORNEUM* | Sweet | Mild | Spleen, Stomach, Small intestine, Bladder | 31 | 1.05% |
| Ezhu | *CURCUMAE RHIZOMA* | Pungent, Bitter | Warm | Liver, Spleen | 29 | 0.98% |
| Zhiqiao | *AURANTII FRUCTUS* | Bitter, Pungent, Sour | Cold | Spleen, Stomach | 26 | 0.88% |
| Honghua | *CARTHAMI FLOS* | Pungent | Warm | Heart, Liver | 25 | 0.85% |
| Huzhang | *POLYGONI CUSPIDATI RHIZOMA ET RADIX* | Bitter | Cold | Liver, Gallbladder, Lung | 25 | 0.85% |
| Dahuang | *RHEI RADIX ET RHIZOMA* | Bitter | Cold | Spleen, Stomach, Large intestine, Liver, Heart包 | 23 | 0.78% |
| Taizishen | *PSEUDOSTELLARIAE RADIX* | Sweet, Bitter | Mild | Spleen, Lung | 23 | 0.78% |
| Yiyiren | *COICIS SEMEN* | Sweet, Weak | Cool | Spleen, Stomach, Lung | 23 | 0.78% |
| Gouqizi | *LYCII FRUCTUS* | Sweet | Mild | Liver, Kidney | 22 | 0.74% |
| San qi | *NOTOGINSENG RADIX ET RHIZOMA* | Sweet, Bitter | Warm | Liver, Stomach | 22 | 0.74% |
| Wuweizi | *SCHISANDRAE CHINENSIS FRUCTUS* | Sour, Sweet | Warm | Lung, Heart, Kidney | 22 | 0.74% |
| Baihuasheshecao | *HERBA HEDYOTIS* | Sweet | Cool | Stomach, Large intestine, Small intestine | 21 | 0.71% |
| Maiya | *HORDEI FRUCTUS GERMINATUS* | Sweet | Mild | Spleen, Stomach | 21 | 0.71% |
| Shanzha | *CRATAEGI FRUCTUS* | Sour, Sweet | Warm | Spleen, Stomach, Liver | 21 | 0.71% |
| Xiangfu | *CYPERI RHIZOMA* | Pungent, Bitter, Sweet | Mild | Liver, Spleen, Tri-jiao | 21 | 0.71% |
| Muli | *OSTREAE CONCHA* | Salty | Cold | Liver, Gallbladder, Kidney | 20 | 0.68% |
| Muxiang | *AUCKLANDIAE RADIX* | Pungent, Bitter | Warm | Spleen, Stomach, Large intestine, Tri-jiao, Gallbladder | 20 | 0.68% |
| San leng | *SPARGANII RHIZOMA* | Pungent, Bitter | Mild | Liver, Spleen | 20 | 0.68% |

**Supplementary File S4. 23 association rules of compensated hepatitis B cirrhosis**

| **Items (Left-hand side => Right-hand side)** | **Support** | **Confidence** | **Lift** |
| --- | --- | --- | --- |
| {Astragali Radix, Paeoniae Radix Alba, Salviae Miltiorrhizae Radix Et Rhizoma} => {Bupleuri Radix} | 16.33% | 100.00% | 1.81 |
| {Atrctylodis Macrocephalae Rhizoma, Poria, Salviae Miltiorrhizae Radix Et Rhizoma} => {Paeoniae Radix Alba} | 21.43% | 95.45% | 1.61 |
| {Paeoniae Radix Alba, Poria, Salviae Miltiorrhizae Radix Et Rhizoma} => {Atrctylodis Macrocephalae Rhizoma} | 21.43% | 95.45% | 1.77 |
| {Bupleuri Radix, Poria, Salviae Miltiorrhizae Radix Et Rhizoma} => {Atrctylodis Macrocephalae Rhizoma} | 18.37% | 94.74% | 1.75 |
| {Bupleuri Radix, Poria, Salviae Miltiorrhizae Radix Et Rhizoma} => {Paeoniae Radix Alba} | 18.37% | 94.74% | 1.6 |
| {Glycyrrhizae Radix Et Rhizoma, Poria} => {Bupleuri Radix} | 17.35% | 94.44% | 1.71 |
| {Glycyrrhizae Radix Et Rhizoma, Poria} => {Atrctylodis Macrocephalae Rhizoma} | 17.35% | 94.44% | 1.75 |
| {Angelicae Sinensis Radix, Poria} => {Atrctylodis Macrocephalae Rhizoma} | 17.35% | 94.44% | 1.75 |
| {Bupleuri Radix, Poria} => {Atrctylodis Macrocephalae Rhizoma} | 34.69% | 94.44% | 1.75 |
| {Atrctylodis Macrocephalae Rhizoma, Bupleuri Radix, Poria, Salviae Miltiorrhizae Radix Et Rhizoma} => {Paeoniae Radix Alba} | 17.35% | 94.44% | 1.6 |
| {Bupleuri Radix, Paeoniae Radix Alba, Poria, Salviae Miltiorrhizae Radix Et Rhizoma} => {Atrctylodis Macrocephalae Rhizoma} | 17.35% | 94.44% | 1.75 |
| {Atrctylodis Macrocephalae Rhizoma, Bupleuri Radix, Paeoniae Radix Alba, Salviae Miltiorrhizae Radix Et Rhizoma} => {Poria} | 17.35% | 94.44% | 1.89 |
| {Bupleuri Radix, Glycyrrhizae Radix Et Rhizoma, Poria} => {Atrctylodis Macrocephalae Rhizoma} | 16.33% | 94.12% | 1.74 |
| {Atrctylodis Macrocephalae Rhizoma, Glycyrrhizae Radix Et Rhizoma, Poria} => {Bupleuri Radix} | 16.33% | 94.12% | 1.71 |
| {Astragali Radix, Atrctylodis Macrocephalae Rhizoma, Bupleuri Radix} => {Salviae Miltiorrhizae Radix Et Rhizoma} | 16.33% | 94.12% | 1.71 |
| {Bupleuri Radix, Paeoniae Radix Alba, Poria} => {Atrctylodis Macrocephalae Rhizoma} | 30.61% | 93.75% | 1.73 |
| {Atrctylodis Macrocephalae Rhizoma, Bupleuri Radix, Paeoniae Radix Alba} => {Poria} | 30.61% | 93.75% | 1.88 |
| {Paeoniae Radix Alba, Poria} => {Atrctylodis Macrocephalae Rhizoma} | 34.69% | 91.89% | 1.7 |
| {Atrctylodis Macrocephalae Rhizoma, Paeoniae Radix Alba} => {Poria} | 34.69% | 91.89% | 1.84 |
| {Atrctylodis Macrocephalae Rhizoma, Paeoniae Radix Alba, Salviae Miltiorrhizae Radix Et Rhizoma} => {Poria} | 21.43% | 91.30% | 1.83 |
| {Astragali Radix, Paeoniae Radix Alba} => {Bupleuri Radix} | 20.41% | 90.91% | 1.65 |
| {Atrctylodis Macrocephalae Rhizoma, Bupleuri Radix, Salviae Miltiorrhizae Radix Et Rhizoma} => {Poria} | 18.37% | 90.00% | 1.8 |
| {Atrctylodis Macrocephalae Rhizoma, Bupleuri Radix, Salviae Miltiorrhizae Radix Et Rhizoma} => {Paeoniae Radix Alba} | 18.37% | 90.00% | 1.52 |

**Supplementary File S5. 42 association rules of decompensated hepatitis B cirrhosis**

| **Items (Left-hand side => Right-hand side)** | **Support** | **Confidence** | **Lift** |
| --- | --- | --- | --- |
| {Plantaginis Semen}=> {Atrctylodis Macrocephalae Rhizoma} | 18.26% | 100.00% | 1.48 |
| {Plantaginis Semen, Poria}=> {Atrctylodis Macrocephalae Rhizoma} | 16.60% | 100.00% | 1.48 |
| {Astragali Radix, Atrctylodis Macrocephalae Rhizoma, Polyporus}=> {Poria} | 16.60% | 100.00% | 1.42 |
| {Arecae Pericarpium, Astragali Radix, Atrctylodis Macrocephalae Rhizoma}=> {Poria} | 22.82% | 98.21% | 1.39 |
| {Alismatis Rhizoma, Astragali Radix, Polyporus}=> {Poria} | 17.01% | 97.62% | 1.38 |
| {Alismatis Rhizoma, Astragali Radix, Atrctylodis Macrocephalae Rhizoma}=> {Poria} | 24.07% | 96.67% | 1.37 |
| {Alismatis Rhizoma, Atrctylodis Macrocephalae Rhizoma, Polyporus}=> {Poria} | 21.16% | 96.23% | 1.36 |
| {Astragali Radix, Polyporus}=> {Poria} | 19.09% | 95.83% | 1.36 |
| {Arecae Pericarpium, Atrctylodis Macrocephalae Rhizoma}=> {Poria} | 26.14% | 95.45% | 1.35 |
| {Astragali Radix, Codonopsis Radix}=> {Atrctylodis Macrocephalae Rhizoma} | 16.18% | 95.12% | 1.41 |
| {Alismatis Rhizoma, Arecae Pericarpium, Atrctylodis Macrocephalae Rhizoma}=> {Poria} | 16.18% | 95.12% | 1.35 |
| {Arecae Pericarpium, Astragali Radix}=> {Poria} | 24.07% | 95.08% | 1.35 |
| {Arecae Pericarpium, Astragali Radix, Poria}=> {Atrctylodis Macrocephalae Rhizoma} | 22.82% | 94.83% | 1.4 |
| {Alismatis Rhizoma, Astragali Radix}=> {Poria} | 27.80% | 94.37% | 1.34 |
| {Astragali Radix, Atrctylodis Macrocephalae Rhizoma, Glycyrrhizae Radix Et Rhizoma}=> {Poria} | 20.75% | 94.34% | 1.34 |
| {Alismatis Rhizoma, Polyporus}=> {Poria} | 26.56% | 94.12% | 1.33 |
| {Alismatis Rhizoma, Atrctylodis Macrocephalae Rhizoma}=> {Poria} | 32.78% | 94.05% | 1.33 |
| {Alismatis Rhizoma, Trionycis Carapax}=> {Poria} | 19.09% | 93.88% | 1.33 |
| {Atrctylodis Macrocephalae Rhizoma, Polyporus}=> {Poria} | 22.82% | 93.22% | 1.32 |
| {Angelicae Sinensis Radix, Astragali Radix, Poria}=> {Atrctylodis Macrocephalae Rhizoma} | 17.01% | 93.18% | 1.38 |
| {Arecae Pericarpium}=> {Poria} | 28.22% | 93.15% | 1.32 |
| {Atrctylodis Macrocephalae Rhizoma, Glycyrrhizae Radix Et Rhizoma}=> {Poria} | 27.80% | 93.06% | 1.32 |
| {Alismatis Rhizoma, Glycyrrhizae Radix Et Rhizoma}=> {Poria} | 16.60% | 93.02% | 1.32 |
| {Alismatis Rhizoma, Arecae Pericarpium, Poria}=> {Atrctylodis Macrocephalae Rhizoma} | 16.18% | 92.86% | 1.37 |
| {Atrctylodis Macrocephalae Rhizoma, Polyporus, Poria}=> {Alismatis Rhizoma} | 21.16% | 92.73% | 2.09 |
| {Arecae Pericarpium, Poria}=> {Atrctylodis Macrocephalae Rhizoma} | 26.14% | 92.65% | 1.37 |
| {Astragali Radix, Glycyrrhizae Radix Et Rhizoma, Poria}=> {Atrctylodis Macrocephalae Rhizoma} | 20.75% | 92.59% | 1.37 |
| {Alismatis Rhizoma}=> {Poria} | 41.08% | 92.52% | 1.31 |
| {Paeoniae Radix Alba}=> {Atrctylodis Macrocephalae Rhizoma} | 19.50% | 92.16% | 1.36 |
| {Codonopsis Radix, Poria}=> {Atrctylodis Macrocephalae Rhizoma} | 18.67% | 91.84% | 1.36 |
| {Arecae Pericarpium, Astragali Radix}=> {Atrctylodis Macrocephalae Rhizoma} | 23.24% | 91.80% | 1.36 |
| {Alismatis Rhizoma, Arecae Pericarpium}=> {Poria} | 17.43% | 91.30% | 1.29 |
| {Alismatis Rhizoma, Atrctylodis Macrocephalae Rhizoma, Salviae Miltiorrhizae Radix Et Rhizoma}=> {Poria} | 17.43% | 91.30% | 1.29 |
| {Alismatis Rhizoma, Astragali Radix, Salviae Miltiorrhizae Radix Et Rhizoma}=> {Poria} | 17.01% | 91.11% | 1.29 |
| {Polyporus}=> {Poria} | 29.46% | 91.03% | 1.29 |
| {Plantaginis Semen}=> {Poria} | 16.60% | 90.91% | 1.29 |
| {Atrctylodis Macrocephalae Rhizoma, Plantaginis Semen}=> {Poria} | 16.60% | 90.91% | 1.29 |
| {Arecae Pericarpium, Salviae Miltiorrhizae Radix Et Rhizoma}=> {Poria} | 16.60% | 90.91% | 1.29 |
| {Poria, Salviae Miltiorrhizae Radix Et Rhizoma, Trionycis Carapax}=> {Atrctylodis Macrocephalae Rhizoma} | 20.75% | 90.91% | 1.34 |
| {Angelicae Sinensis Radix, Atrctylodis Macrocephalae Rhizoma, Salviae Miltiorrhizae Radix Et Rhizoma}=> {Astragali Radix} | 16.18% | 90.70% | 1.33 |
| {Arecae Pericarpium}=> {Atrctylodis Macrocephalae Rhizoma} | 27.39% | 90.41% | 1.34 |
| {Polyporus, Poria}=> {Alismatis Rhizoma} | 26.56% | 90.14% | 2.03 |

**Supplementary File S6. 102 Active Ingredients of Herbs**

| **Herb** | **ID** | **Molecular name** | **OB** | **DL** |
| --- | --- | --- | --- | --- |
| Angelicae Sinensis Radix | MOL000358 | beta-sitosterol | 36.91 | 0.75 |
| Angelicae Sinensis Radix | MOL000449 | Stigmasterol | 43.83 | 0.76 |
| Salviae Miltiorrhizae Radix Et Rhizoma | MOL001601 | 1,2,5,6-tetrahydrotanshinone | 38.75 | 0.36 |
| Salviae Miltiorrhizae Radix Et Rhizoma | MOL001659 | Poriferasterol | 43.83 | 0.76 |
| Salviae Miltiorrhizae Radix Et Rhizoma | MOL001771 | poriferast-5-en-3beta-ol | 36.91 | 0.75 |
| Salviae Miltiorrhizae Radix Et Rhizoma | MOL001942 | isoimperatorin | 45.46 | 0.23 |
| Salviae Miltiorrhizae Radix Et Rhizoma | MOL002222 | sugiol | 36.11 | 0.28 |
| Salviae Miltiorrhizae Radix Et Rhizoma | MOL002651 | Dehydrotanshinone II A | 43.76 | 0.4 |
| Salviae Miltiorrhizae Radix Et Rhizoma | MOL002776 | Baicalin | 40.12 | 0.75 |
| Salviae Miltiorrhizae Radix Et Rhizoma | MOL000569 | digallate | 61.85 | 0.26 |
| Salviae Miltiorrhizae Radix Et Rhizoma | MOL000006 | luteolin | 36.16 | 0.25 |
| Salviae Miltiorrhizae Radix Et Rhizoma | MOL006824 | α-amyrin | 39.51 | 0.76 |
| Salviae Miltiorrhizae Radix Et Rhizoma | MOL007036 | 5,6-dihydroxy-7-isopropyl-1,1-dimethyl-2,3-dihydrophenanthren-4-one | 33.77 | 0.29 |
| Salviae Miltiorrhizae Radix Et Rhizoma | MOL007041 | 2-isopropyl-8-methylphenanthrene-3,4-dione | 40.86 | 0.23 |
| Salviae Miltiorrhizae Radix Et Rhizoma | MOL007045 | 3α-hydroxytanshinoneⅡa | 44.93 | 0.44 |
| Salviae Miltiorrhizae Radix Et Rhizoma | MOL007048 | (E)-3-[2-(3,4-dihydroxyphenyl)-7-hydroxy-benzofuran-4-yl]acrylic acid | 48.24 | 0.31 |
| Salviae Miltiorrhizae Radix Et Rhizoma | MOL007049 | 4-methylenemiltirone | 34.35 | 0.23 |
| Salviae Miltiorrhizae Radix Et Rhizoma | MOL007050 | 2-(4-hydroxy-3-methoxyphenyl)-5-(3-hydroxypropyl)-7-methoxy-3-benzofurancarboxaldehyde | 62.78 | 0.4 |
| Salviae Miltiorrhizae Radix Et Rhizoma | MOL007051 | 6-o-syringyl-8-o-acetyl shanzhiside methyl ester | 46.69 | 0.71 |
| Salviae Miltiorrhizae Radix Et Rhizoma | MOL007058 | formyltanshinone | 73.44 | 0.42 |
| Salviae Miltiorrhizae Radix Et Rhizoma | MOL007059 | 3-beta-Hydroxymethyllenetanshiquinone | 32.16 | 0.41 |
| Salviae Miltiorrhizae Radix Et Rhizoma | MOL007061 | Methylenetanshinquinone | 37.07 | 0.36 |
| Salviae Miltiorrhizae Radix Et Rhizoma | MOL007063 | przewalskin a | 37.11 | 0.65 |
| Salviae Miltiorrhizae Radix Et Rhizoma | MOL007064 | przewalskin b | 110.32 | 0.44 |
| Salviae Miltiorrhizae Radix Et Rhizoma | MOL007068 | Przewaquinone B | 62.24 | 0.41 |
| Salviae Miltiorrhizae Radix Et Rhizoma | MOL007069 | przewaquinone c | 55.74 | 0.4 |
| Salviae Miltiorrhizae Radix Et Rhizoma | MOL007070 | (6S,7R)-6,7-dihydroxy-1,6-dimethyl-8,9-dihydro-7H-naphtho[8,7-g]benzofuran-10,11-dione | 41.31 | 0.45 |
| Salviae Miltiorrhizae Radix Et Rhizoma | MOL007071 | przewaquinone f | 40.31 | 0.46 |
| Salviae Miltiorrhizae Radix Et Rhizoma | MOL007077 | sclareol | 43.67 | 0.21 |
| Salviae Miltiorrhizae Radix Et Rhizoma | MOL007079 | tanshinaldehyde | 52.47 | 0.45 |
| Salviae Miltiorrhizae Radix Et Rhizoma | MOL007081 | Danshenol B | 57.95 | 0.56 |
| Salviae Miltiorrhizae Radix Et Rhizoma | MOL007082 | Danshenol A | 56.97 | 0.52 |
| Salviae Miltiorrhizae Radix Et Rhizoma | MOL007085 | Salvilenone | 30.38 | 0.38 |
| Salviae Miltiorrhizae Radix Et Rhizoma | MOL007088 | cryptotanshinone | 52.34 | 0.4 |
| Salviae Miltiorrhizae Radix Et Rhizoma | MOL007093 | dan-shexinkum d | 38.88 | 0.55 |
| Salviae Miltiorrhizae Radix Et Rhizoma | MOL007094 | danshenspiroketallactone | 50.43 | 0.31 |
| Salviae Miltiorrhizae Radix Et Rhizoma | MOL007098 | deoxyneocryptotanshinone | 49.4 | 0.29 |
| Salviae Miltiorrhizae Radix Et Rhizoma | MOL007100 | dihydrotanshinlactone | 38.68 | 0.32 |
| Salviae Miltiorrhizae Radix Et Rhizoma | MOL007101 | dihydrotanshinoneⅠ | 45.04 | 0.36 |
| Salviae Miltiorrhizae Radix Et Rhizoma | MOL007105 | epidanshenspiroketallactone | 68.27 | 0.31 |
| Salviae Miltiorrhizae Radix Et Rhizoma | MOL007107 | C09092 | 36.07 | 0.25 |
| Salviae Miltiorrhizae Radix Et Rhizoma | MOL007108 | isocryptotanshi-none | 54.98 | 0.39 |
| Salviae Miltiorrhizae Radix Et Rhizoma | MOL007111 | Isotanshinone II | 49.92 | 0.4 |
| Salviae Miltiorrhizae Radix Et Rhizoma | MOL007115 | manool | 45.04 | 0.2 |
| Salviae Miltiorrhizae Radix Et Rhizoma | MOL007118 | microstegiol | 39.61 | 0.28 |
| Salviae Miltiorrhizae Radix Et Rhizoma | MOL007119 | miltionone Ⅰ | 49.68 | 0.32 |
| Salviae Miltiorrhizae Radix Et Rhizoma | MOL007120 | miltionone Ⅱ | 71.03 | 0.44 |
| Salviae Miltiorrhizae Radix Et Rhizoma | MOL007121 | miltipolone | 36.56 | 0.37 |
| Salviae Miltiorrhizae Radix Et Rhizoma | MOL007122 | Miltirone | 38.76 | 0.25 |
| Salviae Miltiorrhizae Radix Et Rhizoma | MOL007123 | miltirone Ⅱ | 44.95 | 0.24 |
| Salviae Miltiorrhizae Radix Et Rhizoma | MOL007124 | neocryptotanshinone ii | 39.46 | 0.23 |
| Salviae Miltiorrhizae Radix Et Rhizoma | MOL007125 | neocryptotanshinone | 52.49 | 0.32 |
| Salviae Miltiorrhizae Radix Et Rhizoma | MOL007127 | 1-methyl-8,9-dihydro-7H-naphtho[5,6-g]benzofuran-6,10,11-trione | 34.72 | 0.37 |
| Salviae Miltiorrhizae Radix Et Rhizoma | MOL007130 | prolithospermic acid | 64.37 | 0.31 |
| Salviae Miltiorrhizae Radix Et Rhizoma | MOL007132 | (2R)-3-(3,4-dihydroxyphenyl)-2-[(Z)-3-(3,4-dihydroxyphenyl)acryloyl]oxy-propionic acid | 109.38 | 0.35 |
| Salviae Miltiorrhizae Radix Et Rhizoma | MOL007141 | salvianolic acid g | 45.56 | 0.61 |
| Salviae Miltiorrhizae Radix Et Rhizoma | MOL007142 | salvianolic acid j | 43.38 | 0.72 |
| Salviae Miltiorrhizae Radix Et Rhizoma | MOL007143 | salvilenone Ⅰ | 32.43 | 0.23 |
| Salviae Miltiorrhizae Radix Et Rhizoma | MOL007145 | salviolone | 31.72 | 0.24 |
| Salviae Miltiorrhizae Radix Et Rhizoma | MOL007150 | (6S)-6-hydroxy-1-methyl-6-methylol-8,9-dihydro-7H-naphtho[8,7-g]benzofuran-10,11-quinone | 75.39 | 0.46 |
| Salviae Miltiorrhizae Radix Et Rhizoma | MOL007151 | Tanshindiol B | 42.67 | 0.45 |
| Salviae Miltiorrhizae Radix Et Rhizoma | MOL007152 | Przewaquinone E | 42.85 | 0.45 |
| Salviae Miltiorrhizae Radix Et Rhizoma | MOL007154 | tanshinone iia | 49.89 | 0.4 |
| Salviae Miltiorrhizae Radix Et Rhizoma | MOL007155 | (6S)-6-(hydroxymethyl)-1,6-dimethyl-8,9-dihydro-7H-naphtho[8,7-g]benzofuran-10,11-dione | 65.26 | 0.45 |
| Salviae Miltiorrhizae Radix Et Rhizoma | MOL007156 | tanshinone Ⅵ | 45.64 | 0.3 |
| Poria | MOL000273 | (2R)-2-[(3S,5R,10S,13R,14R,16R,17R)-3,16-dihydroxy-4,4,10,13,14-pentamethyl-2,3,5,6,12,15,16,17-octahydro-1H-cyclopenta[a]phenanthren-17-yl]-6-methylhept-5-enoic acid | 30.93 | 0.81 |
| Poria | MOL000275 | trametenolic acid | 38.71 | 0.8 |
| Poria | MOL000279 | Cerevisterol | 37.96 | 0.77 |
| Poria | MOL000282 | ergosta-7,22E-dien-3beta-ol | 43.51 | 0.72 |
| Poria | MOL000283 | Ergosterol peroxide | 40.36 | 0.81 |
| Poria | MOL000287 | 3beta-Hydroxy-24-methylene-8-lanostene-21-oic acid | 38.7 | 0.81 |
| Poria | MOL000296 | hederagenin | 36.91 | 0.75 |
| Paeoniae Radix Alba | MOL000359 | sitosterol | 36.91 | 0.75 |
| Paeoniae Radix Alba | MOL000358 | beta-sitosterol | 36.91 | 0.75 |
| Paeoniae Radix Alba | MOL000422 | kaempferol | 41.88 | 0.24 |
| Paeoniae Radix Alba | MOL001919 | (3S,5R,8R,9R,10S,14S)-3,17-dihydroxy-4,4,8,10,14-pentamethyl-2,3,5,6,7,9-hexahydro-1H-cyclopenta[a]phenanthrene-15,16-dione | 43.56 | 0.53 |
| Paeoniae Radix Alba | MOL001924 | paeoniflorin | 53.87 | 0.79 |
| Paeoniae Radix Alba | MOL000492 | (+)-catechin | 54.83 | 0.24 |
| Paeoniae Radix Alba | MOL000211 | Mairin | 55.38 | 0.78 |
| Paeoniae Radix Alba | MOL001918 | paeoniflorgenone | 87.59 | 0.37 |
| Astragali Radix | MOL000211 | Mairin | 55.38 | 0.78 |
| Astragali Radix | MOL000239 | Jaranol | 50.83 | 0.29 |
| Astragali Radix | MOL000296 | hederagenin | 36.91 | 0.75 |
| Astragali Radix | MOL000033 | (3S,8S,9S,10R,13R,14S,17R)-10,13-dimethyl-17-[(2R,5S)-5-propan-2-yloctan-2-yl]-2,3,4,7,8,9,11,12,14,15,16,17-dodecahydro-1H-cyclopenta[a]phenanthren-3-ol | 36.23 | 0.78 |
| Astragali Radix | MOL000354 | isorhamnetin | 49.6 | 0.31 |
| Astragali Radix | MOL000371 | 3,9-di-O-methylnissolin | 53.74 | 0.48 |
| Astragali Radix | MOL000378 | 7-O-methylisomucronulatol | 74.69 | 0.3 |
| Astragali Radix | MOL000379 | 9,10-dimethoxypterocarpan-3-O-β-D-glucoside | 36.74 | 0.92 |
| Astragali Radix | MOL000380 | (6aR,11aR)-9,10-dimethoxy-6a,11a-dihydro-6H-benzofurano[3,2-c]chromen-3-ol | 64.26 | 0.42 |
| Astragali Radix | MOL000387 | Bifendate | 31.1 | 0.67 |
| Astragali Radix | MOL000392 | formononetin | 69.67 | 0.21 |
| Astragali Radix | MOL000417 | Calycosin | 47.75 | 0.24 |
| Astragali Radix | MOL000422 | kaempferol | 41.88 | 0.24 |
| Astragali Radix | MOL000433 | FA | 68.96 | 0.71 |
| Astragali Radix | MOL000438 | (3R)-3-(2-hydroxy-3,4-dimethoxyphenyl)chroman-7-ol | 67.67 | 0.26 |
| Astragali Radix | MOL000439 | isomucronulatol-7,2'-di-O-glucosiole | 49.28 | 0.62 |
| Astragali Radix | MOL000442 | 1,7-Dihydroxy-3,9-dimethoxy pterocarpene | 39.05 | 0.48 |
| Astragali Radix | MOL000098 | quercetin | 46.43 | 0.28 |
| Atrctylodis Macrocephalae Rhizoma | MOL000022 | 14-acetyl-12-senecioyl-2E,8Z,10E-atractylentriol | 63.37 | 0.3 |
| Atrctylodis Macrocephalae Rhizoma | MOL000033 | (3S,8S,9S,10R,13R,14S,17R)-10,13-dimethyl-17-[(2R,5S)-5-propan-2-yloctan-2-yl]-2,3,4,7,8,9,11,12,14,15,16,17-dodecahydro-1H-cyclopenta[a]phenanthren-3-ol | 36.23 | 0.78 |
| Atrctylodis Macrocephalae Rhizoma | MOL000049 | 3β-acetoxyatractylone | 54.07 | 0.22 |
| Atrctylodis Macrocephalae Rhizoma | MOL000072 | 8β-ethoxy atractylenolide Ⅲ | 35.95 | 0.21 |
